# Endothelial CDS2 deficiency causes VEGFA-mediated vascular regression and tumor inhibition

**DOI:** 10.1038/s41422-019-0229-5

**Published:** 2019-09-09

**Authors:** Wencao Zhao, Le Cao, Hanru Ying, Wenjuan Zhang, Dantong Li, Xiaolong Zhu, Wenzhi Xue, Shuang Wu, Mengye Cao, Cong Fu, Haonan Qi, Yimei Hao, Yun-Chi Tang, Jun Qin, Tao P. Zhong, Xiaoxi Lin, Luyang Yu, Xuri Li, Lin Li, Dianqing Wu, Weijun Pan

**Affiliations:** 1 Key Laboratory of Tissue Microenvironment and Tumor, CAS Center for Excellence in Molecular Cell Science, Shanghai Institute of Nutrition and Health, University of Chinese Academy of Sciences, Chinese Academy of Sciences (CAS), Shanghai, China; 2 Department of Plastic and Reconstructive Surgery, Shanghai Ninth People’s Hospital, Shanghai Jiaotong University, School of Medicine, Shanghai, China; 3 Shanghai Key Laboratory of Regulatory Biology, Institute of Molecular Medicine, East China Normal University School of Life Sciences, Shanghai, China; 4 Innovative Research Team of High-level Local University in Shanghai, Shanghai, China; 5 Institute of Genetics, College of Life Sciences, Zhejiang University, Hangzhou, China; 6 State Key Laboratory of Ophthalmology, Zhongshan Ophthalmic Center, Sun Yat-sen University, Guangzhou, China; 7 State Key Laboratory of Molecular Biology, CAS Center for Excellence in Molecular Cell Science, Institute of Biochemistry and Cell Biology, Shanghai Institutes for Biological Sciences, CAS, Shanghai, China; 8 Department of Pharmacology, Vascular Biology and Therapeutic Program, School of Medicine, Yale University, New Haven, CT USA

**Keywords:** Tumour angiogenesis, Growth factor signalling

## Abstract

The response of endothelial cells to signaling stimulation is critical for vascular morphogenesis, homeostasis and function. Vascular endothelial growth factor-a (VEGFA) has been commonly recognized as a pro-angiogenic factor in vertebrate developmental, physiological and pathological conditions for decades. Here we report a novel finding that genetic ablation of CDP-diacylglycerol synthetase-2 (CDS2), a metabolic enzyme that controls phosphoinositide recycling, switches the output of VEGFA signaling from promoting angiogenesis to unexpectedly inducing vessel regression. Live imaging analysis uncovered the presence of reverse migration of the angiogenic endothelium in *cds2* mutant zebrafish upon VEGFA stimulation, and endothelium regression also occurred in postnatal retina and implanted tumor models in mice. In tumor models, CDS2 deficiency enhanced the level of tumor-secreted VEGFA, which in-turn trapped tumors into a VEGFA-induced vessel regression situation, leading to suppression of tumor growth. Mechanistically, VEGFA stimulation reduced phosphatidylinositol (4,5)-bisphosphate (PIP2) availability in the absence of CDS2-controlled-phosphoinositide metabolism, subsequently causing phosphatidylinositol (3,4,5)-triphosphate (PIP3) deficiency and FOXO1 activation to trigger regression of CDS2-null endothelium. Thus, our data indicate that the effect of VEGFA on vasculature is context-dependent and can be converted from angiogenesis to vascular regression.

## Introduction

The blood vessel system, consisting of arteries, veins and interconnecting capillaries, can deliver oxygen, nutrients, metabolites and carry circulating blood cells to support human life. Vessels interweaved within tissues also coordinate developmental organogenesis, regulate injury repair and regeneration, and form niches for adult stem cells^1–5^. Vascular homeostasis is maintained mainly through its remodeling, including growth and pruning/regression. Dysregulation or dysfunction of the vascular system is associated with numerous types of human diseases^6^.

Vascular growth is mainly governed by angiogenesis, a well-studied process of new blood vessel formation from the existing vessels, and vasculogenesis, the de novo formation of blood vessels using stem cells, involving extracellular matrix remodeling, tip or stalk cell fate determination, endothelial cell migration, proliferation and the subsequent stabilization by mural cells^7^. This process is managed by numerous angiogenic signaling factors such as VEGFs, FGF2, PDGFs, etc.^8,9^. VEGFA, a well-known pro-angiogenic factor, governs vasculogenesis and angiogenesis and maintains vascular stability in embryonic development, physiological and pathological conditions. Activation of VEGFA signaling leads to activation of downstream signal arms, including PLCγ and PI3K, both of which utilize membrane lipid-phosphatidylinositol-4,5-bisphosphate (PIP2) to mediate signal transduction and exert their angiogenic effects^10^.

As important as vasculogenesis and angiogenesis, vessel regression also plays important roles in embryonic development and tissue homeostasis. It governs the elimination of hyaloid vessels^11^ and the maturation of retinal and brain vasculature^12–14^. Vascular regression also occurs in the adult luteolysis and couples with breast endothelium degeneration after lactation^15,16^. Mechanistically, vessel regression is regulated by hemodynamics and numerous signaling pathways^13^. Generally, lack of VEGFA in tissues is an important reason for the cease of blood flow, endothelial apoptosis and subsequent regression of endothelium^17–20^. Besides, DLL4/Notch signaling activation promotes retinal vessel regression by inducing vasoconstriction and interrupting blood flow^21^, and loss of Wnt signaling transducers (β-catenin, Lef1, Evi/Wls or Rspo3) results in reduction of retinal endothelial cell proliferation and blood vessel regression^22–24^.

CDP-diacylglycerol (CDP-DAG) synthase-2 (CDS2), an enzyme utilizing phosphatidic acid (PA) to produce CDP-DAG, is responsible for the recycling of phospholipids, including phosphatidylglycerol (PG), phosphatidylinositol (PI) and their derivates, cardiolipin (CL), PIP2 and phosphatidylinositol (3,4,5)-triphosphate (PIP3)^25,26^. Disruption of CDS in photoreceptor cells of *Drosophila* limits PIP2 availability and PLC-mediated signaling, leading to a decreased amplitude of light response^26^. Loss of CDS also reduces PI and PI-derived PIP3 levels, resulting in accumulation of neutral lipids along with reduced cell and organ size in the *Drosophila*^25^. Vertebrates have two CDS genes, *CDS1* and *CDS2*, both of which regulate the growth of lipid droplets and adipocyte development in cultured human cells^27^. Our previous study in zebrafish model shows that *cds2*, with enriched mRNA expression in endothelium, plays an important role in vascular morphogenesis. *cds2* mutants show reduced VEGFA signaling activity and inefficient angiogenesis phenotype, which can be rescued by delivery of PIP2-containg liposome, suggesting that the recycling of phosphoinositides is essential for angiogenesis^28^.

In this study, we report an unexpected observation that VEGFA stimulation promotes vessel regression in CDS2-deficient endothelia. Without the CDS2-controlled phosphoinositide metabolic circuits, the VEGFA-PLCγ signaling axis hydrolyzes PIP2, leading to depletion of PIP3 and FOXO1 nucleus accumulation/activation to trigger reverse migration of angiogenic endothelium. Importantly, these observations and mechanisms are conserved in both zebrafish and mice. Thus, the outcome of VEGFA stimulation may switch from promoting neovascularization to inducing vascular regression, which depends on the specific endothelial metabolic status and signaling crosstalk.

## Results

### VEGFA triggers vessel regression in *cds2*-deficient zebrafish embryos

**Fig. 1:**
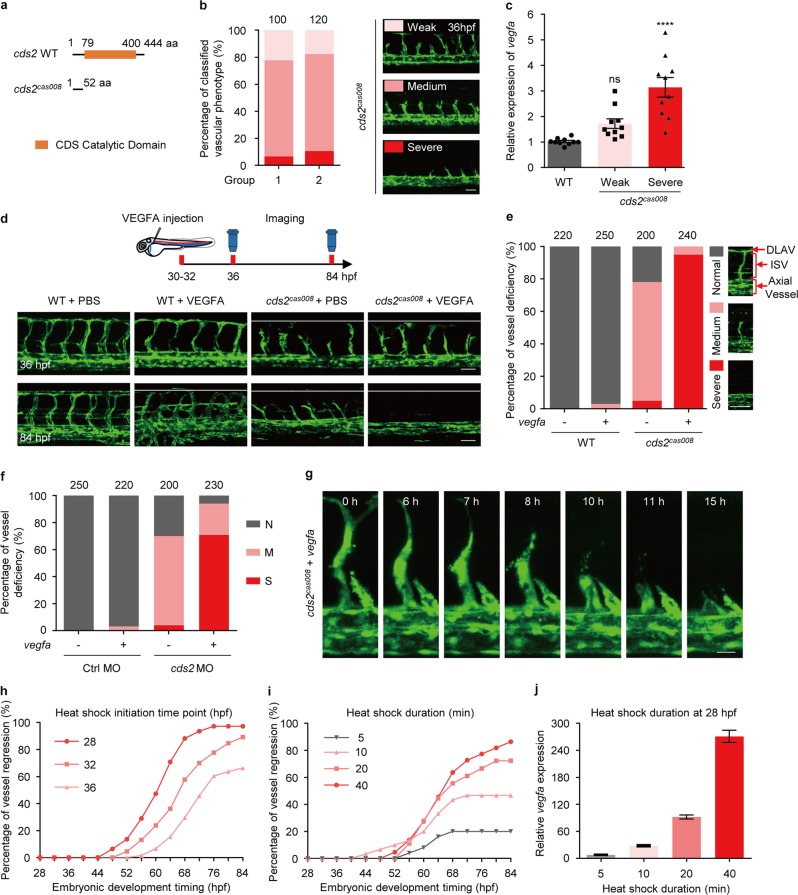
VEGFA induces vessel regression in *cds2-*deficient zebrafish. **a** Diagram of zebrafish *cds2* mutant allele cas008 (*cds2*^*cas008*^). **b** Quantification of classified vascular phenotypes in *cds2* mutant embryos at 36 hpf. The right panel shows the representative images of weak, medium and severe vascular defects in *cds2* mutant embryos within *Tg*(*fli1a:eGFP*) transgenic background. The counted embryo number is shown on the top of bar graph. **c** Relative *vegfa* expression in 36 hpf WT and *cds2* mutant embryos within two phenotypic subtypes. *n* *=* 10 samples per group, 10 embryos pooled for each sample. **d** Confocal images of trunk vessels from 36 and 84 hpf WT and *cds2* mutant embryos injected with VEGFA protein or PBS. White lines indicate the dorsal position where ISVs in WT embryos can reach. The top panel represents timing for venous microinjection (CCV) and confocal imaging analysis. **e** Quantification of vascular deficiency in the embryos (Fig. S1**e**) at 84 hpf. Bars show percentage of classified ISVs that have grown to the dorsal trunk (Normal), to the myoseptum (Medium) or regressed to the axial vessels (Severe). Representative images for 3 classified phenotypes are shown on the right. The counted ISV number from 20–25 embryos per group (10 trunk segments counted per embryo) is shown on the top bar graph. DLAV, dorsal longitudinal anastomotic vessel. **f** Quantitative analysis of trunk vessels in WT and *cds2* MO-injected embryos with or w/o *vegfa* OE. The quantified ISV number shown on the top graph is from 20–25 embryos per group. N, normal; M, medium; S, severe. **g** Time-lapse imaging analysis on *cds2* mutants with *Tg(fli1a:eGFP)* transgenic background and *vegfa* OE during 52–72 hpf, showing ISV regression process. **h**, **i** Time-lapse phenotype analysis on vessel regression events under different heat shock initiation time points (**h**, embryos were heat-shocked for 1 h at 28, 32 or 36 hpf, *n* = 300 ISVs counted from 30 embryos) and various heat shock durations (**i**, *n* = 300 ISVs counted from 30 embryos). **j** Relative *vegfa* expression of *Tg(hsp:vegfaa*) embryos challenged with different heat shock durations. *vegfa* level was determined at 2–4 h after heat shock induction and normalized to wild type embryos under the same experimental conditions. *n* = 4 samples per group, 20 embryos pooled for each sample. Scale bars, 50 μm (**b** and **d**; 36 hpf), 70 μm (**d**; 84 hpf), 100 μm (**e**) and 20 μm (**g**). Error bars, mean ± SEM. Statistical significance between the indicated sample versus WT is ****P < 0.0001 or ns, not significant (*P* ≥ 0.05). See also Supplementary information, Fig. S1

We previously reported that zebrafish *cds2* mutants carried endothelium-specific morphogenic defects with impaired angiogenesis of intersegmental vessel (ISV) and reduced VEGFA signal activity^28^. During the phenotype scoring on zebrafish *cds2* mutants (Fig. 1a, b), we found there were always about 5–10% embryos showing more severe, while about 15–20% showing less severe vascular defects, compared to the majority of *cds2* mutants (Fig. 1b). After checking the expression level of *vegfaa* (the major *vegfa* isoform in zebrafish embryos that governs vasculogenesis and angiogenesis)^29–31^, we found these *cds2* mutants with severe vascular defects expressed a higher level of *vegfaa*, compared to those with a weaker phenotype (Fig. 1c), which is opposite to our expectation. We further analyzed Hif1a pathway and found that Hif1a protein was accumulated and its downstream signaling was activated in *cds2* mutants with severe defective vessel phenotype (Supplementary information, Fig. S1a, b), indicating hypoxia might cause upregulation of *vegfaa*.

To understand whether increased VEGFA level correlates with the severity of vascular defects and explore the role of VEGFA in the vascular morphogenesis in *cds2* mutants, we injected purified recombinant VEGFA protein into the circulation of *cds2* mutants (with normal or weaker phenotype) (Fig. 1d). Interestingly, almost all ISVs in VEGFA-injected *cds2* mutants disappeared in 50 h (Fig. 1d). To further confirm this phenomenon with statistical analysis, *cds2* mutants were crossed into *Tg(hsp:vegfaa)* transgenic background^32^. After heat shock induction, *vegfaa* expression reached a peak level in 4 h and then remained at least 5 folds higher compared to that in siblings without VEGFA induction (Supplementary information, Fig. S1c). *vegfa* over-expression (OE) caused hyper-branching in wildtype siblings (Supplementary information, Fig. S1d, e), however, over 90% of the *cds2* mutants displayed complete loss of ISVs in their trunk regions at 84 h post fertilization (hpf), which was over 50 h post heat shock induction of *vegfa* (Fig. 1e). This was about the same time at which VEGFA protein injection caused the phenotype (Fig. 1d and Supplementary information, Fig. S1e). We also tested *cds2* morphants, which phenocopied *cds2* mutants showing VEGFA-induced loss of angiogenic vasculature (Supplementary information, Fig. S1f, g, Fig. 1f).

Live imaging analysis revealed that ISVs in *cds2* mutants initiated reverse migration at about 16 h post heat shock induction of *vegfaa* and about 65% endothelial cells underwent apoptosis before merging into axial vessels (Fig. 1g, Supplementary information, Fig. S1h, Video S1, 2 and 4), while EC proliferation barely happened in this process compared with that in normal angiogenesis (Supplementary information, Video S3 and S4). Time-lapse scoring of vascular phenotype also confirmed that vessel regression initiated at 16–20 h post heat shock induction and took about 40 h in-total to reach over 80% regression (Fig. 1h and Supplementary information, Fig. S1i). The duration of vessel regression remained unchanged when we sequentially delayed the heat shock induction time points and the percentage of ISV regression depended on *vegfa* expression level (Fig. 1h–j). The morphology of axial vessels, including dorsal aorta and cardinal vein, remained intact during ISV regression. In addition, we found that regression of ISV also occurred in zebrafish *cds2* mutants without *vegfa* OE (Supplementary information, Video S2). Such regression occurred slowly and stopped around 52 hpf, probably due to moderate stimulation of ventral (lower level) to dorsal (higher level) gradient of transiently expressed endogenous *vegfa*^33^.

### Vessel regression in endothelium-specific *Cds2* knockout mice

**Fig. 2:**
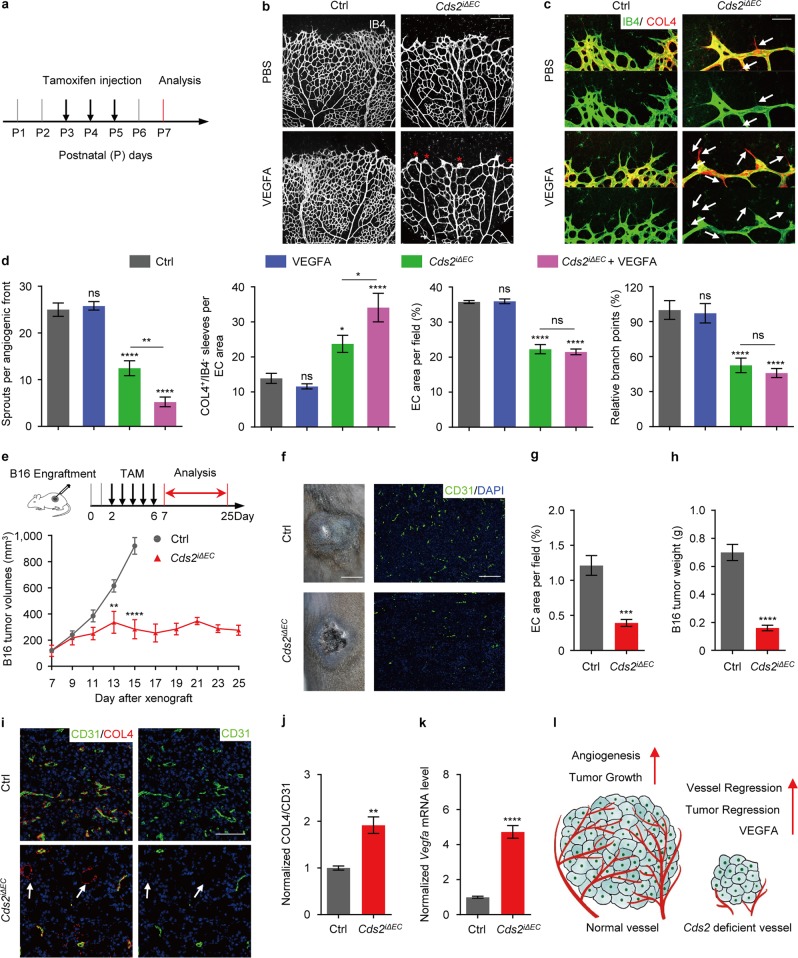
Vessel regression in CDS2 endothelium-specific knockout mice. **a** Model of tamoxifen treatment on mouse pups. Confocal images of isolectin B4 (IB4) (**b**) and IB4/Collagen IV (COL4) (**c**) double stained retinal vessels at postnatal day (P) 7 in *Cds2*^*iΔEC*^ or control mice with or w/o VEGFA injection. Asterisks display the blunt angiogenic front. Arrows indicate COL4^+^/IB4^−^ empty sleeves. **d** Bar graphs showing analysis of angiogenic sprouts, COL4^+^/IB4^−^ empty sleeves, endothelial area and vascular branch points. *n* = 6–10 mice per group. Inactivation of *Cds2* caused retardation of B16 tumor growth (**e**), necrosis (**f**; left panels, day11 tumors), blood vessel (**f**; right panels and **g**) and tumor weight (**h**) reduction. Schematic diagram (**e**) shows the strategy to inactivate *Cds2* after tumor cell implantation. *n* = 6–8 tumors from 6–8 mice per group. Anti-COL4 and anti-CD31 co-immunostaining (**i**) and quantitative analysis (**j**) on B16 tumors from control or *Cds2*^*iΔEC*^ mice at 13 days post-implantation. Arrows indicate regressed vessels. *n* = 6 tumors from 6 mice as a group. **k** Quantification of *Vegfa* mRNA level in day 9 B16 tumors. *n* = 8 tumors from 4 mice per group. **l** Model of VEGFA dose-dependent tumor vessel regression in CDS2-deficient endothelium. Left panel shows that increased angiogenesis associates with increased tumor growth in WT mice. Right panel depicts that vessel regression occurs in CDS2-deficient vessels, associated with high level of VEGFA produced by tumor, which in turn further advances vessel regression and blocks tumor progression. Scale bars, 200 μm (**b** and **f**; right panel), 70 μm (**c**), 0.5 cm (**f**; left panel) and 100 μm (**i**). Error bars, mean ± SEM. Statistical significance between the indicated sample versus control or between the marked pairs are *P < 0.05, **P < 0.01, ***P < 0.001, ****P < 0.0001, or ns, not significant (*P* ≥ 0.05). See also Supplementary information, Figs. S2–S5

The observed VEGFA-induced vessel regression has never been reported before. We therefore set to verify whether it is conserved in vertebrates, especially in mammals. Endothelial-specific tamoxifen-inducible *Cds2* knockout mice (*Cds2*^*iΔEC*^) were generated by putting *loxP* sites on both side of *exon2* in *Cds2* locus and crossing the floxed *Cds2* mice into *Cdh5:Cre-ERT2* transgenic background^34^ (Supplementary information, Fig. S2a, b). Significant downregulation of *Cds2* was validated in the endothelium of *Cds2*^*iΔEC*^ mice. We also found that the expression of *Cds2* was highly enriched in endothelium in wildtype mice (Supplementary information, Fig. S2c, d). First of all, we performed phenotypic analysis on retinal vasculature (Fig. 2a–d), a system that has been well established for studying vascular morphogenesis^35^. *Cds2*^*iΔEC*^ caused significant reduction in endothelium coverage and the number of branch points, compared to those of retinal vasculature in the control mice (Fig. 2b, d). Furthermore, there were significantly reduced endothelial sprouts, but increased Collagen 4 (COL4)-positive and isolectin B4 (IB4)-negative sleeves (an indication of regressed vessel) in the angiogenic front of retinal vasculature in *Cds2*^*iΔEC*^ mice (Fig. 2c, d). Importantly, as observed in zebrafish *cds2* mutants, this defective vascular morphogenesis in *Cds2*^*iΔEC*^ mice could be further enhanced by injection of recombinant VEGFA at postnatal day (P) 4 and 5, which resulted in blunted angiogenic front with further reduced endothelial sprouts (Fig. 2a–d). We next assessed whether cell apoptosis and proliferation were altered in *Cds2*^*iΔEC*^ mice. In line with zebrafish study, endothelial apoptosis was also observed in the retinal vasculature of *Cds2*^*iΔEC*^ mice (Supplementary information, Fig. S2e, f), and a reduction of EC proliferation in *Cds2*^*iΔEC*^ mice was revealed by EdU incorporation, which was further declined by VEGFA stimulation (Supplementary information, Fig. S2g, h).

VEGFA has been reported to govern vasculogenesis and angiogenesis in most of developmental, physiological and pathological processes, including tumor angiogenesis^9,36,37^. The B16 melanoma or TC-1 lung carcinoma cells have been reported to progress in an angiogenesis-dependent manner^38–40^. Interestingly, we found that either B16 or TC-1 cells could barely form tumors after implantation into the *Cds2*^*iΔEC*^ mice, whereas they formed tumors readily in the control mice (Supplementary information, Fig. S3a–j). Further analysis showed that there were dramatically reduced vascular densities inside those tiny tumors harvested from the *Cds2*^*iΔEC*^ mice, compared to those from the control mice (Supplementary information, Fig. S3a–j).

To further examine the vascular dynamics in the anti-tumor role of CDS2 deficiency, endothelium-specific *Cds2* knockout was induced by tamoxifen injection after tumor cell implantation (Fig. 2e and Supplementary information, Fig. S4a). These tumors could grow as rapidly in the initial 7 days as those in the control mice, but then grew slower and even regressed in *Cds2*^*iΔEC*^ mice (Fig. 2e and Supplementary information, Fig. S4a). We tracked B16 tumor growth retardation in *Cds2*^*iΔEC*^ mice up to 35 days without finding resistance or reoccurrence, while most of B16 tumors in control mice reached the size limitation (1000 mm^3^, allowed by Institute Animal Research Protocol) within 18 days (Fig. 2e). More interestingly, all TC-1 tumors were dramatically regressed in *Cds2*^*iΔEC*^ KO mice and finally disappeared in 21–25 days, without reoccurrence up to 35 days (Supplementary information, Fig. S4a, b), and we did observe high frequent tumor necrosis in both B16 and TC-1 tumors (Fig. 2f and Supplementary information, Fig. S4c). Immunostaining analysis showed severely reduced vascular density inside the tumors, which highly correlated with reduced tumor weight (Fig. 2f–h and Supplementary information, Fig. S4c, d). In addition, more endothelium regression (COL4-positive/CD31-negative sleeves) was observed in retarded B16 tumors in *Cds2*^*iΔEC*^ mice (Fig. 2i, j). Because TC-1 tumor cells themselves expressed high level of COL4, ultrasound imaging assay showed gradually reduced blood flow inside TC-1 tumors after endothelium-specific *Cds2* KO in the recipient mice (Supplementary information, Fig. S4e).

After checking the expression level of *Vegfa* in these tumors, like we did in zebrafish *cds2* mutants, we found that both B16 and TC-1 tumors, harvested from *Cds2*^*iΔEC*^ mice at 9 days post-implantation, expressed much higher *Vegfa* than tumors harvested from the control mice, although the vasculature inside these *Cds2*^*iΔEC*^ tumors had dramatically regressed (Fig. 2k and Supplementary information, Fig. S4f). Thus, increased VEGFA seems not to promote angiogenesis inside *Cds2*^*iΔEC*^ tumors, but correlates with vessel regression as observed in zebrafish *cds2* mutants (Fig. 1 and Supplementary information, Fig. S1). As expected, tumor cells in *Cds2*^*iΔEC*^ mice were more hypoxic than those in control mice and expressed much higher VEGFA compared to non-tumor cells (Supplementary information, Fig. S4g–i). Therefore, we hypothesized that, once tumors reached certain size, hypoxia would drive VEGFA expression which would normally trigger angiogenesis, but caused vessel regression in the absence of *Cds2*. Reduced or reversed angiogenesis would further enhance the hypoxia condition, which subsequently caused more VEGFA expression and triggered the greater vessel regression, eventually leading to retarded growth or regression of the tumors (Fig. 2l).

Intriguingly, *Cds2*^*iΔEC*^ mice were viable during all experiments and vasculature in organs, including heart, kidney and liver, was intact (Supplementary information, Fig. S5a, b), which might be due to the highly expressed VEGFA in *Cds2*^*iΔEC*^ tumors compared to these organs (Supplementary information, Fig. S5c). Beyond VEGFA, the expression levels of other angiogenic ligands, including FGF2, PIGF and PDGFB were also upregulated (Supplementary information, Fig. S5d), which was commonly seen in tumors with anti-VEGFA antibody or anti-VEGFR2 inhibitor administration for rapid development of resistance. However, we did not observe any resistance or reoccurrence of tumor in *Cds2*^*iΔEC*^ mice. Microinjection of ectopic FGF2 also failed to rescue ISV defects in *cds2* mutant zebrafish with *vegfa* OE, although it is sufficient to cause hyper-branching ISV phenotype in control zebrafish embryos (Supplementary information, Fig. S5e, f).

### PIP3 metabolic exhaustion governs VEGFA-induced vessel regression

**Fig. 3:**
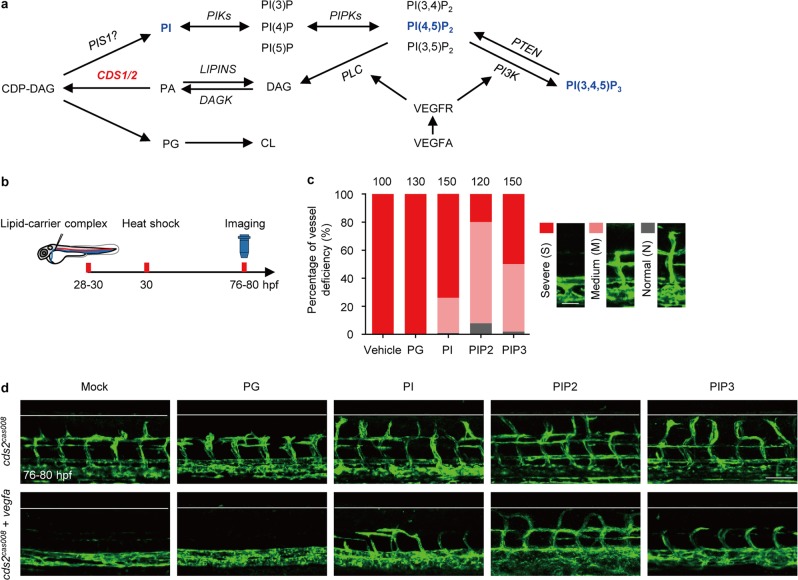
PI-PIPs metabolism is involved in CDS2-deficient vessel regression. **a** Schematic diagram of CDS2-controlled phosphoinositide recycling. DAG, diacylglycerol; PA, phosphatidic acid; PIS1, phosphatidylinositol synthase 1; PI, phosphoinositol; PIK, phosphoinositol 3/4/5-kinase; PIPK, phosphatidylinositol 4/5-phosphate 5/4-kinase; PTEN, phosphatase and tensin homolog; PI3K, phosphoinositide 3 kinase; PLC, phospholipase c; PG, phosphatidylglycerol; CL, cardiolipin. **b** Model of timing for phospholipid-carrier mixture microinjection, heatshock induction and confocal imaging analysis in (**c**) and (**d**). **c** Quantification of rescue effects of different phospholipids (PI, PG, PIP2 and PIP3) on *vegfa* OE-induced ISV regression in *cds2* mutant embryos. Bars show percentages of vessel deficiency. The representative images of classified vascular phenotype are shown on the right. The counted ISV numbers were shown on the top, 10 ISV per embryo. **d** Representative images of trunk vessels from *cds2* mutants at 76–80 hpf with or w/o *vegfa* OE and with microinjection of lipid–carrier complex, including PG, PI, PIP2 or PIP3 at 28–30 hpf. Scale bars, 50 μm (**c**) and 100 μm (**d**)

To explore the mechanism by which CDS2 deficiency converts VEGFA signaling from pro-angiogenic effect to promoting vessel regression, we carried out liposome rescue experiments on *cds2* zebrafish mutants with *vegfa* OE. CDP-DAG, the product of CDS2^25,26^, could be further converted to either PG or PI to supply PIPs and CL, both of which have been reported to play important roles as cell signaling messengers or regulators^41–45^ (Fig. 3a). We found that microinjection of liposomes, carrying synthetic PI, its derivates PI(4,5)P_2_ (PIP2) or PI(3,4,5)P_3_ (PIP3), but not the PG-containing liposome, could significantly rescue the formation of ISV sprouts in *vegfa*-OE *cds2* mutants (Fig. 3b–d).

**Fig. 4:**
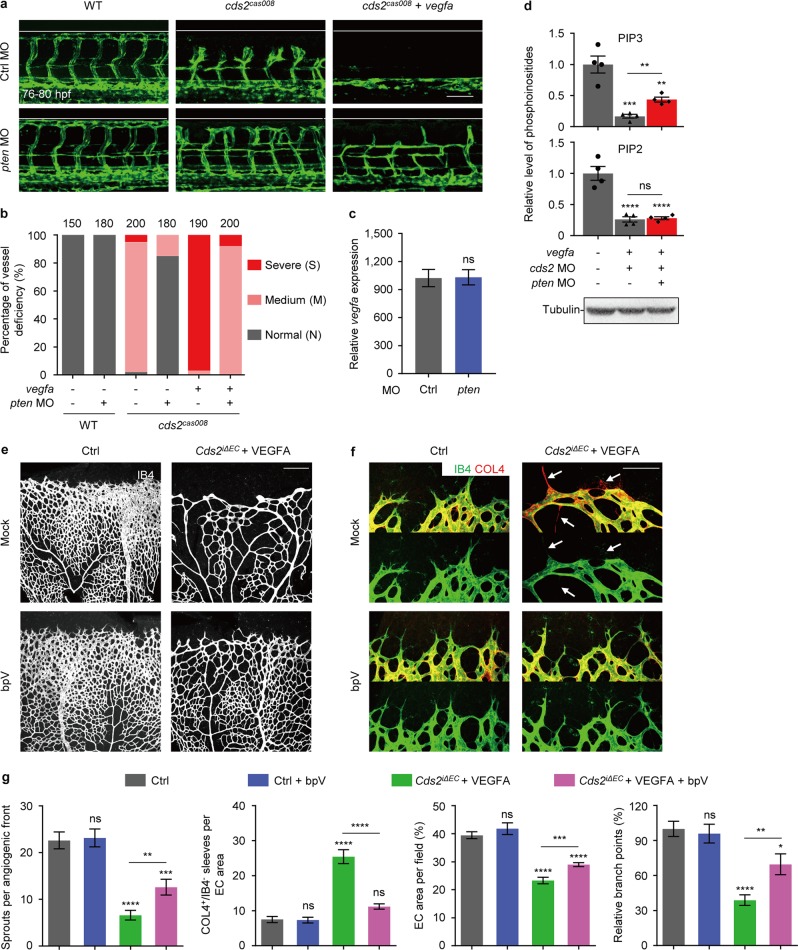
PIP3 exhaustion governs VEGFA-induced regression of CDS2-deficient endothelium. Confocal images (**a**) and quantitative analysis (**b**) of vessel-deficient phenotype in WT zebrafish embryos or *cds2* mutants with or w/o *vegfa* OE, with control MO or *pten* MO (combination of *ptena* and *ptenb* MO) injection. The counted ISV number shown on the top (**b**) is from 15–20 embryos per group. **c** Relative *vegfa* mRNA level of *cds2* mutants with *vegfa* OE injected with ctrl or *pten* MO. The expression was normalized to WT embryos. *n* = 3 samples per group, 15–20 embryos pooled for each sample. **d** *pten* knockdown partially restored PIP3, but not PIP2 level in *cds2*-deficient endothelium with *vegfa* OE. Western blotting analysis on a-Tubulin serves as the internal control for cell amounts in each sample collected during lipid quantitation analysis. *n* = 4 samples each group. Confocal images (**e** and **f**) and quantitative analysis (**g**) of P7 retinal vessels stained by IB4 (**e**) and IB4/COL4 (**f**) from control or VEGFA-injected *Cds2*^*iΔEC*^ mice treated with bpV (PTEN inhibitor) or vehicle (saline). bpV (2 mg kg^−1^) or vehicle was given three times at P2, P4 and P6. Arrows show regressed vessels. Angiogenic sprouts, COL4^+^/IB4^−^ empty sleeves, endothelial area and branch points were quantified in (**g**), *n* = 6–10 mice per group. Scale bars, 100 μm (**a** and **f**) and 200 μm (**e**). Error bars, mean ± SEM. *P < 0.05; **P < 0.01; ***P < 0.001; ****P < 0.0001; ns, not significant (*P* ≥ 0.05). See also Supplementary information, Fig. S6

To distinguish the role of PIP2 and PIP3 in VEGFA-induced vessel regression in *cds2* mutants, we knocked down PTEN to block the conversion of PIP3 back to PIP2^46,47^, and found PTEN inhibition could significantly rescue the vascular defects in *cds2* mutants with or without *vegfa* OE, without affecting *vegfa* OE level (Fig. 4a–c). Lipid quantitation showed that VEGFA stimulation indeed caused dramatic reduction of PIP2 and PIP3 in CDS2-deficient endothelium, and PTEN knockdown rescued the level of PIP3, but not PIP2, in CDS2-deficient endothelium under VEGFA stimulation (Fig. 4d and Supplementary information, Fig. S5g). In addition, we found inhibition of PTEN through small molecular compound, bpV^48,49^, could also rescue vascular defects in retinal vasculature of *Cds2*^*iΔEC*^ mice even with VEGFA stimulation, by increasing the number of angiogenic sprouts and reducing vessel regression (Fig. 4e–g). These results indicate that the availability of PIP3 plays a key role in VEGFA-induced regression of CDS2-deficient endothelium.

Vessel regression or inefficient angiogenesis has been linked to hyper-activation of Notch signaling^21,50,51^, inactivation of Wnt/β-catenin or silencing of Ca^2^^+^ signaling^22–24,52^. To test whether these signal pathways are involved in VEGFA-induced vessel regression, we firstly treated *vegfa*-OE *cds2* mutants with Notch inhibitor, DAPT. *vegfa* OE indeed activated Notch signaling, which could be completely abolished by DAPT treatment (Supplementary information, Fig. S6a). However, vessel regression persisted in *cds2* mutants with *vegfa* OE (Supplementary information, Fig. S6b). Furthermore, neither activation of Wnt/β-catenin signaling (endothelium-specific expression of constitutively activated form of β-catenin)^53^ nor activation of Ca^2^^+^ signaling (Inomycin treatment)^54,55^ could rescue vessel regression phenotype in *cds2* mutants with *vegfa* OE (Supplementary information, Fig. S6c–f). In addition, blood flow in axial vessels remained normal during ISV reverse migration of *cds2*-deficient embryos with *vegfa* OE and pericyte or smooth muscle cell coverage of the vessels occurred later than vessel regression in zebrafish embryogenesis^56^, so the possibilities that ISV regression caused by blood flow blockage or abnormal coverage of mural cells^12,14,57^ are very unlikely.

### FOXO1 activation in VEGFA-induced CDS2-deficient endothelium regression

**Fig. 5:**
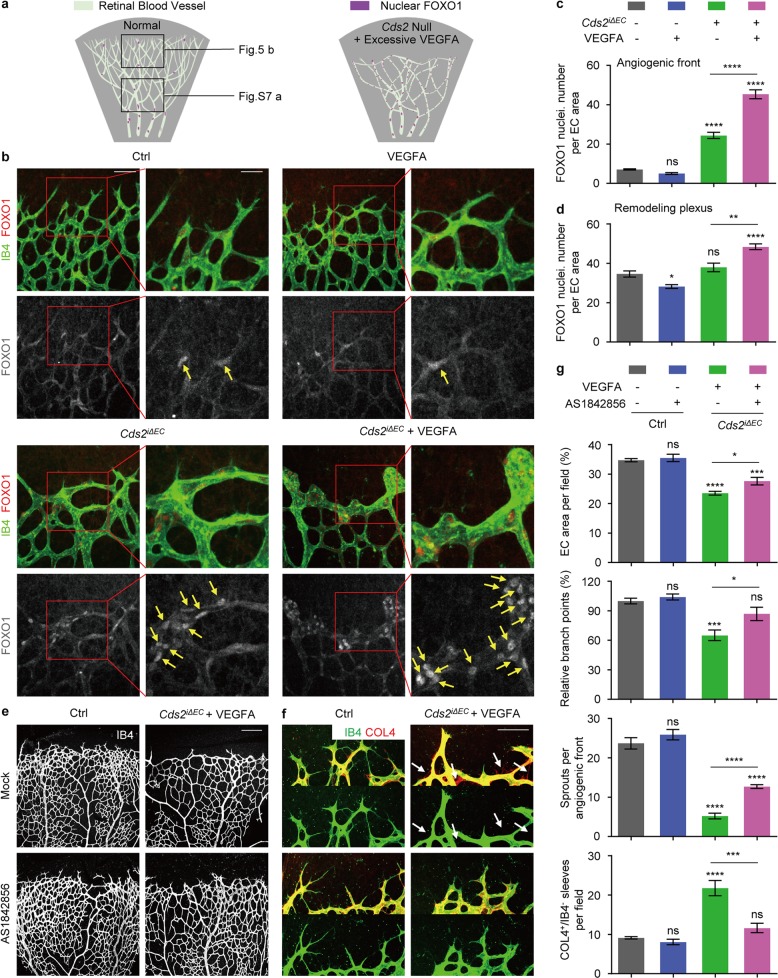
FOXO1 activation is required for VEGFA-induced vessel regression in mouse retina model. **a** Schematic diagram showing nucleus FOXO1 distribution in retinal vasculature. In WT, nucleus FOXO1 mainly locate in remodeling endothelial plexus, however they are gathering to the angiogenic front of CDS2-deficient endothelium, which could be further increased by excessive VEGFA stimulation. IB4 and FOXO1 co-immunostaining (**b**) and quantitative analysis (**c**) on P7 retinas in control and *Cds2*^*iΔEC*^ mice with or w/o recombinant VEGFA stimulation. Yellow arrows indicate nuclear localization of FOXO1. *n* = 8 mice per group. **d** Quantification of IB4 and FOXO1 co-staining of P7 retinal vessels in the remodeling plexus area from WT or *Cds2*^*iΔEC*^ embryos with or without ectopic VEGFA injection. *n* = 5–8 mice per group. Confocal imaging analysis of P7 retinal vessels stained by IB4 (**e**) and IB4/COL4 (**f**) from control or VEGFA-injected *Cds2*^*iΔEC*^ mice treated with AS1842856 (FOXO1 inhibitor) or vehicle. Arrows show regressed vessels. **g** Quantitative analysis on endothelial area, branch points, angiogenic sprouts and COL4^+^/IB4^−^ empty sleeves of P7 retinal vessels in control and VEGFA-injected *Cds2*^*iΔEC*^ mice with or without AS1842856 treatment. *n* = 6–10 mice per group. Scale bars, 50 μm (**b**; left panel), 25 μm (**b**; magnification figure), 200 μm (**e**) and 100 μm (**f**). Error bars, mean ± SEM. *P < 0.05; **P < 0.01 ***P < 0.001; ****P < 0.0001; ns, not significant (*P* ≥ 0.05). See also Supplementary information, Fig. S7

FOXO1 has been reported to govern angiogenesis by regulating endothelium metabolism and apoptosis^48,58,59^, and it is negatively regulated by PIP3/Akt signaling through limiting its nuclear translocation and hence transcription activity^60–62^. Endothelium-specific knockout of *Foxo1* causes hyper-branching of retinal vasculature, while overexpression of constitutively activated FOXO1 (AKT phosphorylation sites are mutated, constitutive nuclear localization) causes deficient vascular morphogenesis^58^, which is similar to that observed in the retinal vasculature of *Cds2*^*iΔEC*^ mice (Fig. 2b, d). Immunostaining analysis on retinal vasculature showed that, nuclear FOXO1 mainly appeared in the remodeling plexus region, but not the angiogenic front in the retinal vasculature in the control mice, which was consistent to previous reports^58^ (Fig. 5a–d and Supplementary information, Fig. S7a).

We found, different from that in the control mice, FOXO1 protein predominantly located in endothelial nuclei in the front area of retinal vasculature in *Cds2*^*iΔEC*^ mice (3.4 folds increase, compared to Control), which could be further enhanced by additional VEGFA stimulation (9.1 folds increase, compared to control with recombinant VEGFA stimulation) that resulted in dramatically increased nucleus FOXO1 in blunted angiogenic front with significantly reduced sprouts (Fig. 5a–c). This FOXO1 activation was further validated by transcript analysis of FOXO1 target genes in retinal endothelial cells (Supplementary information, Fig. S7b)^58^. To examine whether activated FOXO1 signaling governs vessel regression in *Cds2*^*iΔEC*^ mice, we applied the treatment of FOXO1-specific small molecular inhibitor, AS1842856 (AS), in the same experiments^48^. We found that AS treatment could significantly increase the endothelium coverage, the number of branch points and the angiogenic sprouts in the retinal vasculature of *Cds2*^*iΔEC*^ mice (Fig. 5e–g). Specifically, COL4-positive/IB4 negative sleeves, remained by vessel regression, was remarkably reduced in the frontier retinal *Cds2*^*iΔEC*^ vasculature even with ectopic VEGFA injection (Fig. 5f, g), indicative of the essential role of FOXO1 activation in VEGFA-induced vessel regression of CDS2-deficient endothelium.

**Fig. 6:**
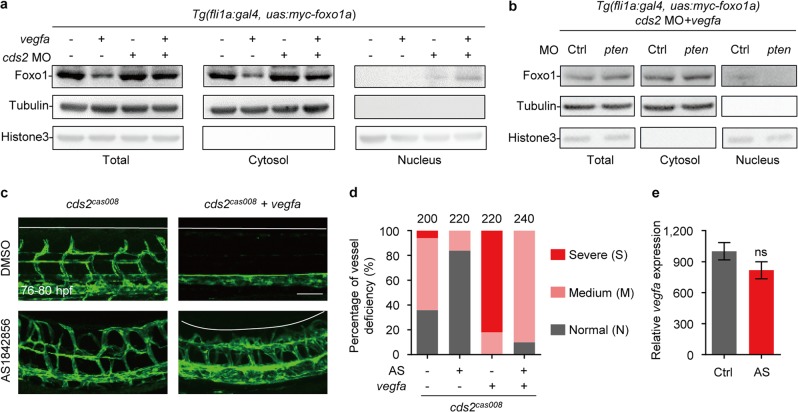
FOXO1 activation is required for VEGFA-induced regression in zebrafish *cds2* mutants. **a** Western blotting analysis shows that Foxo1a translocates to the nuclei in *cds2*-deficient zebrafish endothelium upon VEGFA stimulation. Protein samples for whole embryos or cytosol/nucleus fractions were collected at 48 hpf from *Tg(fli1a:gal4)* embryos with *Tol2 transposase-mediated Tg(uas:myc-foxo1a)* transgenesis, with or w/o *cds2* MO and with or w/o *vegfa* OE *(*heat-shock induction at 28 hpf) as indicated in the figure. **b** Western blotting analysis shows that *pten* knockdown blocks Foxo1a nuclear accumulation in *cds2*-deficient zebrafish endothelium with *vegfa* OE. Protein samples for total/cytosol/nucleus fractions were collected at 48 hpf from *cds2* MO-injected *Tg(fli1a:gal4)* embryos with *Tol2 transposase-mediated Tg(uas:myc-foxo1a)* transgenesis and *vegfa* OE, with or w/o *pten* MO. Representative images (**c**) and phenotype quantitative analysis (**d**) of trunk vessels in *cds2* mutant embryos with or w/o *vegfa* OE (heat-shock induction at 28 hpf) and with or w/o AS1842856 treatment (from 24 hpf) at 76–80 hpf. AS, AS1842856. The ISV number quantified on the top (**d**) is from 20–24 embryos per group. **e** Relative *vegfa* mRNA level in *cds2* mutants with heatshock-induced *vegfa* OE, treated with DMSO or AS. The *vegfa* expression level was determined at 2 h post heat shock induction and normalized to WT embryos without *vegfa* OE. Ctrl, DMSO; AS, AS1842856. *n* = 3 samples, 20 embryos pooled for each sample. Scale bar, 100 μm. Error bar, mean ± SEM. ns, not significant (*P* ≥ 0.05)

Furthermore, consistent with that in mice model, FOXO1 nuclear translocation also occurred in the endothelium of zebrafish *cds2* mutants, but not in wildtype siblings even with VEGFA stimulation (Fig. 6a), which is further supported by reduced phospho-FOXO1 level in CDS2-deficient HUVECs (Supplementary information, Fig. S7c). The level of endothelial nuclear FOXO1 in *cds2* mutants could be enhanced by VEGFA stimulation (Fig. 6a), but rescued by PTEN knockdown (Fig. 6b and Supplementary information, Fig. S7d, e), which further indicated that the FOXO1 nuclear translocation and activation was under the control of PIP3 availability upon VEGFA stimulation (Fig. 4). In addition, AS treatment could also successfully rescue ISV angiogenic sprouts in *cds2* mutants even with *vegfa* OE, although angiogenic front was disorganized, most likely due to the loss of PIP3 (Fig. 6c–e). Thus, we conclude that VEGFA can trigger the regression of *cds2-null* vessels in a FOXO1-dependent manner, which should be blocked by the PI3K-PIP3-Akt axis of VEGFA signaling in normal angiogenesis.

### PIP3 exhaustion as the result of PLCγ-mediated PIP2 hydrolysis in VEGFA-stimulated CDS2-deficient endothelium

**Fig. 7:**
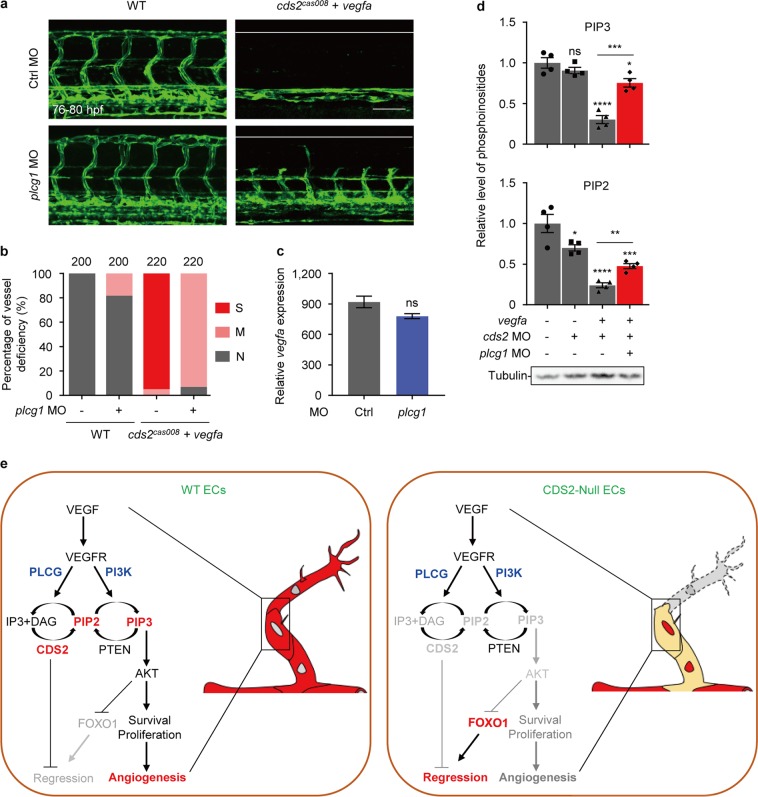
PIP3 reduction is mainly caused by PLCγ mediated PIP2 hydrolysis. Representative confocal images (**a**) and quantitative analysis (**b**) of trunk vessel phenotypes of WT or *cds2* mutant embryos with *vegfa* OE and with or w/o *plcg1* MO. The ISV number counted from 20–22 embryos per group is shown on the top (**b**). **c** Relative *vegfa* mRNA level of *cds2* mutants with *vegfa* OE injected with ctrl or *plcg1* MO. *vegfa* expression level was determined at 2 h post heatshock induction and normalized to WT embryos without *vegfa* OE. *n* = 3 samples, 15–20 embryos pooled for each sample. **d** *plcg1* knockdown partially restored PIP2 and PIP3 level in *cds2*-deficient endothelium with *vegfa* OE. Western blotting analysis on a-Tubulin serves as the internal control for cell amounts in each sample collected during lipid quantitation analysis. *n* = 4 samples per group. **e** Working model of VEGFA-triggered vessel regression on CDS2-deficient endothelium. The outcome of VEGFA signaling can be reversed from angiogenesis to vessel regression, which is dependent on CDS2-controlled PIP2 and PIP3 availability and FOXO1 signaling activation. Scale bar, 100 μm. Error bars, mean ± SEM. *P < 0.05; **P < 0.01; ***P < 0.001; ****P < 0.0001; ns, not significant (*P* ≥ 0.05). See also Supplementary information, Fig. S8

To understand how VEGFA stimulation triggers PIP3 reduction in endothelium of CDS2-deficient zebrafish embryos, we tested another signaling arm of VEGFA transduced by PLCγ. PLCγ hydrolyzes PIP2 to produce DAG and IP3, both of which are second messengers for signal transduction to stimulate angiogenesis and will be recycled in a CDS2-mediated PI metabolic circuit to supply the pool of phosphatidylinositides^26^. PIP2 level is relative abundant, compared to that of PIP3^63^, thus PLCγ might serve as a major pathway to consume PIP2. We found partial knockdown of PLCγ (0.5 ng morpholino is not sufficient to block vasculogenesis or angiogenesis) could block vessel regression in *cds2* mutants with *vegfa* OE, without affecting the level of *vegfa* OE induction (Fig. 7a–c). And lipid quantitation also showed that VEGFA-triggered PIP3 and PIP2 reduction in *cds2*-deficient endothelial cells could indeed be rescued by PLCγ knockdown (Fig. 7d). These results indicate that, without CDS2-mediated phosphoinositide recycling, activation of PLCγ signaling by VEGFA causes PIP2 hydrolysis and its eventual reduction, which subsequently causes the reduction of the PIP3 level, leading to FOXO1 activation and vessel regression, as summarized in Fig. 7e.

## Discussion

Homeostasis and remodeling of blood vessels interweaved within tissues and organs are tightly controlled by intercellular metabolism and intracellular signaling network^7,64^, the crosstalking among which determines the specificity and flexibility of final outputs. It has been documented that VEGFB, a close family member to VEGFA, is a context-dependent cytokine. It works as a pro-angiogenic factor in inefficient angiogenesis, but attenuates excessive vascular branches to stabilize vasculature^65^. Here, we reported that the outcome of VEGFA could be altered from pro-angiogenesis to vessel regression when CDS2-dependent phosphoinositide recycling was blocked. Furthermore, we found that VEGFA-induced vessel regression occurred mainly in the angiogenic front of retina and in growing tumors, but not in other vasculature-enriched normal organs, including heart, liver or kidney in *Cds2*^*iΔEC*^ mice (Supplementary information, Fig. S5a, b). We hypothesized that the endothelium under angiogenic status might require more PIP3 availability to balance VEGFA downstream signaling and be more sensitive to FOXO1 activation than those vessels that had been connected to each other or well-covered by pericytes/smooth muscle cells, and might be in quiescent or different metabolic status^58^.

Although the phenotype and causal mechanism of VEGFA-induced vessel regression reported in this study are novel, a number of questions still need to be addressed in future studies. First of all, vertebrate CDS1 and CDS2 share very similar catalytic domains and play similar roles in lipid metabolism regarding energy storage^27^. However, ectopic expression of CDS1 could not rescue vascular phenotype in CDS2-deficient zebrafish embryos either with or without *vegfa* OE. On the other hand, FGF2 also utilizes PLCγ and PI3K to transduce signals, ectopic FGF2 protein injection is sufficient to induce ISV hyper-branching phenotype in WT embryos, but failed to trigger vessel regression in CDS2-deficient zebrafish embryos. Thus, mechanistic interaction between CDS2-controlled phosphoinositide recycling and VEGFA signaling remains elusive.

Our new data argue that PIP3, but not PIP2^28,66^, equilibrates the angiogenic status of endothelial cells upon VEGFA stimulation. However, treatment of PI3K inhibitors on zebrafish embryos (28–76 hpf) did not cause severe ISV regression as CDS2 deficiency did (Supplementary information, Fig. S8a, b), although they were sufficient to block angiogenesis if administration was applied (20–32 hpf) as previously reported^67^ (Supplementary information, Fig. S8c, d). In B16 tumor model, loss of CDS2 showed much more potency to cause tumor inhibition with vascular defects, especially vessel regression, compared to that of PI3K inhibitor (BEZ235 and BKM120) administration (Supplementary information, Fig. S8e–h). In other word, CDS2 inhibition, as a novel multi-targeting approach, could work more efficient than PI3K inhibitors on vessel regression under microenvironmental VEGFA stimulation. This might be due to multiple effects caused by the disruption of phospholipid recycling upon CDS2 deficiency. Actually, deprivation of CDS2 was also reported to lead to phosphatidic acid (PA) accumulation^27^, which served as an important second messenger for signaling transduction^68–70^. Thus, combined effect of upregulated PA and inhibited PIP3 on vascular morphogenesis should be tested under VEGFA stimulation in future studies.

Phosphoinositide recycling is generally considered as a housekeeping metabolic circuit, and currently, there is no commercially available inhibitor to modulate CDS2 activity. In implanted tumor models, the levels of tumor-secreted VEGFA are very high, compared to those in normal tissue or organs. Such expression could be further increased by 4–10 folds in *Cds2*^*iΔEC*^ mice (Fig. 2k and Supplementary information, Fig. S4f). After retro-orbital or intravenous injection of cell-permeable CDS2-specfiic vivo-morpholinos (chemical-modified morpholino with cell penetrating capability) into mice with implanted B16 tumors, they worked efficiently to trigger vessel regression and block tumor progression as that occurred in *Cds2* endothelium-specific knockout mice (Supplementary information, Fig. S8i–k). However, these reagents could only be used as CDS2 inhibitors for research purpose, a CDS2-specific inhibitory small molecular compound will be helpful in future mechanistic characterization and translational applications.

A variety of studies demonstrate that an increased VEGF level usually accompanies with impaired angiogenic activity in diabetes^71–75^. Although a chronic and sustained stimulation of upregulated VEGFA was proposed to be a causal effect, we think that this hypothesis remains controversial since upregulated VEGFA can also result in diabetic retinopathy by excessive angiogenesis^76^. In addition, impaired PI3K-Akt signaling was reported in the myocardium of type 2 diabetic patients with chronic coronary heart disease, coupling with upregulated VEGFA^71^. Elevated VEGFA165 was also reported to exacerbate human type-2 diabetic nephropathy^77^, and administration of VEGFA neutralizing antibody resulted in amelioration of long-term renal changes in obese type-2 diabetic mice^78^. Thus, it will be interesting to know whether upregulated VEGFA associates with deficient phosphoinositide recycling in diabetic condition in heart or kidney, and tissue/organ specific signaling crosstalking and metabolic status must be considered to elucidate the underlying mechanism.

Given that numerous types of cells can secrete VEGFA to induce or regulate vascular niche in various physiological and pathological conditions, including adult stem cells, progenitors, immune cells and others^79–84^, the vascular defects, previously described as impaired angiogenesis, may need to be revisited from the angle of vessel regression by checking microenvironmental VEGFA, phosphoinositides availability, PIP3-Akt activation and FOXO1 nucleus accumulation.

## Material and methods

### Zebrafish husbandry and transgenic lines

All zebrafish studies in this work were performed according to the guidelines of the Animal Ethics Committee and Institutional Animal Care and Use Committee of Shanghai Institutes for Biological Sciences, Chinese Academy of Sciences (Shanghai, China). Zebrafish maintenance and breeding were performed by standard methods^85^. The transgenic line *Tg(fli1a:eGFP)*, *Tg(hsp:vegfaa)*, *Tg(fli1a:gal4)*, *Tg(tp1:dsGFP)*, *Tg(HuC:gal4)*, *Tg(fli1a:ngfp)* and *Tg(uas:GCaMP5)* were described previously^14,32,86–88^. Most experiments in this study were carried out in the embryos generated by outcross of *Tg(hsp:vegfaa)* with other lines if possible. The *cds2* mutant was generated by CRISPR/Cas9 mutagenesis (Supplementary information, Table S1). The heterozygotes or homozygotes were identified by genomic PCR, followed by sequencing. All PCR Oligos was listed in Table S1.

### Microinjection

Morpholino oligos (MOs) were purchased from Gene Tools Company and dissolved in nuclease free water. Embryos were injected at one-cell stage with 1–6 ng control MO, 1 ng *cds2* MO^28^, 0.5 ng *plcg1* MO^89^, 6 ng *ptena* MO^90^, 6 ng *ptenb* MO^90^ or a mixture of MOs if required. The MO sequences was listed in Table S1. For *pTol2:flk1-ΔN β-catenin-2a-mcherry* rescue experiments, embryos were injected with 50 pg of *pTol2:flk1-ΔN β-catenin-2a-mcherry* construct^53^ together with 10 pg *transposase* mRNA. *transposase* mRNA was synthesized in vitro by SP6 mMessage mMachine Transcription Kit (Ambion).

For protein delivery into circulation, 1–2 nl VEGF-A (Sino Biological, 11066-HNAH-5; 250 μg ml^−1^) or FGF2 (Novoprotein, C044; 200 μg ml^−1^) dissolved in water was injected into the common cardinal vein (CCV) of the embryos at 28–32 hpf. For phospholipid delivery, phosphatidylinositol (PI) (Echelon Biosciences, P-0004; 1 mg ml^−1^), phosphatidylglycerol (PG) (Sigma, P8318; 2.5 mg ml^−1^), phosphatidylinositol 3, 4, 5-trisphosphate (PIP3) (Echelon Biosciences, P-3904; 1 mg ml^−1^) and phosphatidylinositol 4, 5-bisphosphate (PIP2) (Echelon Biosciences, P-9045; 1 mg ml^−1^) were dissolved in water and incubated with equal molar concentration of histone H1 carrier (Echelon Biosciences, P-9C2) for 10 min at room temperature before injection. Then, 1–2 nl lipid–carrier mixture were delivered into the CCV of *cds2* mutants or control embryos at 28–30 hpf. Histone H1 carrier alone-injected embryos were used as controls. Vessel regression assessments were done at 76–84 hpf.

### Heatshock induction and compound treatment on zebrafish model

For heat-shock induction, zebrafish embryos in *Tg(hsp:vegfaa)* background were heat-shocked at 28–36 hpf at 37 °C for 1 h and then analyzed for ISV phenotype at the indicated stages. For *vegfa* expression level determination, total RNA from the heat-shocked embryos (10–20 embryos pooled for each sample) at 30–32 hpf was extracted and reverse transcribed to cDNA for qPCR analysis.

For small molecular compound treatments, all chemicals were dissolved in DMSO and diluted in egg water containing 0.045% 1-phenyl-2-thiourea (PTU, Sigma) at the indicated concentrations, including DAPT^67^ (Selleck; 30 μM), AS1842856 (Selleck; 20 nM), Ionomycin (Sigma; 500 nM), BKM120 (TargetMol; 5 μM) and BEZ235 (MCE; 10 μM). Embryos were manually dechorionated and incubated with chemicals at the indicated stage until analysis. To validate the function of ionomycin on zebrafish, embryos were treated at 30 hpf for 10 min and then mounted for confocal imaging analysis.

### Zebrafish TUNEL assay

Zebrafish embryos were collected at 54–60 hpf and fixed with 4% PFA. After methanol dehydration, rehydration, proteinase K digestion and acetone treatment, the embryos were permeabilized in incubating buffer (0.1% Triton X-100, 0.1% sodium citrate in PBS) for 30 min at RT. Then TUNEL staining was performed using the In Situ Cell Death Detection Kit TMR red (Roche) as the manufacturer’s instruction. After that, the embryos were incubated with mouse anti-GFP antibody (Abmart, M20004, 1:500) followed by incubation of Alexa-Fluor 488-conjugated secondary antibody.

### Imaging analysis on live zebrafish embryos

Confocal images of zebrafish live transgenic embryos were obtained by a Zeiss LSM 710 inverted confocal microscope (Carl Zeiss, Germany) or Olympus FV1000 scanning confocal microscope. Live embryos were anesthetized with 0.03% Tricaine (Sigma) and then mounted in 1% low-melt agarose. Images were taken using a Plan Apochromat 10× objective or UPLSAPO 20× objective with 488 nm and/or 559 nm lasers. For time-lapse imaging, embryos were anesthetized with 0.03% Tricaine (Sigma-Aldrich), mounted with 1% low-melt agarose in custom-built chamber and imaged using a Zeiss LSM 880 upright confocal microscope (Carl Zeiss, Germany) equipped with a 20× or 40× objective. Z-stacks were acquired at 2–3 μm increments every 10–14 min. Images were processed by ImageJ (NIH) and Imaris (Bitplane) software.

### Lipid quantification

Endothelial cells were sorted by FACS from *Tg(fli1a:eGFP)* background embryos (2000 per sample) at 48 hpf (before vessel regression), followed by lipid extraction as previously described^91^. In brief, cells were lysed in 3.75 volumes methanol:chloroform:HCl (40:20:1) mixture on ice, followed with 1 volume chloroform and 2.25 volumes water. After vortexing for 1 min, samples were centrifuged at 3000 rpm for 2 min at 4 °C, and the lower organic phase was collected and dried under nitrogen stream. Quantitative analysis on PIP2 or PIP3 was performed by commercial available kits (Echelon Biosciences, K-4500 and K-2500s), with synthetic PIP2 or PIP3 (Echelon Biosciences) as standard control.

### Genetic manipulation and husbandry of mice model

All mice were maintained under specific pathogen-free conditions and handled according to the Animal Ethics Committee and Institutional Animal Care and Use Committee of Shanghai Institutes for Biological Sciences, Chinese Academy of Sciences (Shanghai, China). Project license of mice study used in this work was SIBS-2017-PWJ-1. Floxed Cds2 mice were commercially generated (Shanghai Model Organisms Center). *Cds2*^*fl*/*fl*^ mice were bred into *Cdh5-CreERT2* mice^34^ to generate inducible knockout line in the endothelium. All genotypings were determined by PCR analyses with oligos listed in Table S1.

Cre activation in newborn mice was induced by three consecutive intraperitoneal injections of 50 μl tamoxifen (Sigma, T5648; 2 mg ml^−1^) on postnatal day (P) 3-P5. Injections of vehicle or 1 μg mVEGF-A (Sino Biological, 50159-MNAB; 100 μg ml^−1^) dissolved in PBS were administrated subcutaneously in the region surrounding the eye at P4 and P5. Retina vasculature was analyzed at P7. Tamoxifen-injected *Cre*^−^ littermate animals were used as control. To characterize the efficiency of mouse endothelium-specific *Cds2* knockout, endothelial cell isolation was performed as previously described^92^. In brief, organs were harvested, cut into small pieces and dissociated into single cell suspension by a digestion with 1 mg ml^−1^ collagenase/dispase (Roche, 10269638001). Then cell suspension was incubated with anti-CD31 antibody (BD Biosciences, 553370) pre-incubated microbeads (Miltenyi Biotec) for endothelial cell separation by manufacturer’s protocol. Total RNA of endothelial cells was extracted and reversely transcribed into cDNA. *Cds2* expression relative to the housekeeping gene *Actb* was determined by qPCR.

### Compound treatment on mice retina model

For pharmacological inhibition experiments in mouse pups, pilot experiments were performed to estimate the optimum concentration of each small molecular compound. To inhibit FOXO1 or PTEN, AS1842856 (Calbiochem, 344355; 2.5 mg kg^−1^) or bpV (EMD Millipore, 203701; 2 mg kg^−1^) were injected subcutaneously in the region surrounding the eye at P2, P4 and P6 before dissection at P7. DMSO (5%) or saline was used as vehicle and experimental control.

### Cell culture and manipulation

Mouse lung carcinoma cell line TC-1 and melanoma cell line B16 were purchased from the cell bank of Shanghai Institutes for Biological Sciences. These lines are not listed in the International Cell Line Authentication Committee database. GFP-labeled B16 cells were generated by retroviral transduction with GFP-expressing construct as previously reported^93^. Blasticidin was used for stable cell line selection. TC-1 and B16 cells were cultured in RPMI-1640 and DMEM respectively, supplemented with 10% FBS, 1% Penicillin/Streptomycin. HUVECs (PromoCell) were cultured in endothelial growth medium-2 (PromoCell, C-22011) on plates pre-coated with 1% collagen (Corning, 354236). HUVECs less than passage 5 were used in experiments and tested negative for mycoplasma. CDS2 knockdown was performed as previously described^28^. All cells were tested negative for mycoplasma contamination before experiments by PCR. Primer sequences for this test can be found in Table S1.

### Tumor xenografts

For tumor angiogenesis study, mice aged between 8–12 weeks were injected with 80 mg per kg (weight) tamoxifen intraperitoneally, once daily for 5 days to induce *Cds2* deletion before tumor cell inoculations. Cells were harvested in 100 μl RPMI-1640 or DMEM medium and gently mixed 2:1 (volume ratio) with matrigel (Corning, 356237). Then, mice were inoculated subcutaneously on the lateral flanks with 10^6^ TC-1 cells or 0.5 million B16 cells (150 μl volume per injection). Vernier caliper was used to measure the approximate tumor volume calculated as length × width^2^/2, and final tumor weight measurements were taken at the termination of the experiments. Subsequently, tumors as well as other organs were harvested for histology analysis.

For vessel regression determination, mice were injected with 3 × 10^6^ TC-1 cells or 10^6^ B16 cells (150 μl volume per injection) two days prior to a five-day consecutive injection of tamoxifen, allowing for a robust angiogenic response. Tumors were measured once two days for 25 days or until reaching an average diameter of 1.5 cm, and tumor necrosis was monitored daily during this process. For TC-1 tumors, ultrasound imaging and analysis were performed at day 7, 11 and 15 post-implantation to assess the blood perfusion. Meanwhile, a portion of tumors without necrosis were excised at day 9 or 13 for tumor weight measurements, histology and qRT-PCR analysis. To determine the *Vegfa* expression in tumor cells, GFP-labeled B16 cells were inoculated as described above. Then day 9 tumors were excised, cut into pieces and digested with collagenase/dispase (Roche, 1 mg ml^−1^) in DMEM at 37 °C for 30 min. GFP-positive and -negative cells were sorted by FACS to perform qPCR analysis. To pharmacologically inhibit PI3K signaling, BKM120 or BEZ235 dissolved in 10% N-methyl-2-pyrrolidone (NMP) with 90% PEG400 was orally administrated at 40 mg kg^−1^ daily from day 5 to day 9 after B16 cell inoculation. Then, tumors were excised at day 10 for weight measurement and immunohistochemistry analysis. For vivo morpholino (vMO) administration, *Cds2* vMOs (Gene Tools) were administrated at 1 mg kg^−1^ daily through intravenous injection from day 1 post B16 cell implantation until the experimental termination. Tumor size was measured once two days for 15 days or until reaching size limitation guided by Animal Protocol.

### Ultrasound imaging and analysis

Ultrasound imaging was performed using the Vevo 2100 high-frequency ultrasound system (VisualSonics, Toronto, ON, Canada). Tumor-bearing mice were anesthetized with isoflurane at 1.5% concentration delivered with medical air through a vaporizer. Mice were positioned on a heated platform (THM 150; Indus Instruments, Webster, TX). A solid state transducer (MS-250) was placed on the tumor and held in position by a clamp mounted on the Vevo Rail System. 21-MHz B-mode imaging was used to identify lesions suspicious for cancer (hypoechoic foci), and once a lesion was identified, the 21-MHz transducer was fixed into position on the imaging platform and switched with the 18-MHz transducer for nonlinear contrast-enhanced imaging. Nonlinear Contrast Mode imaging was employed to detect the presence of Vevo MicroMarker^®^ Non-Targeted Contrast Agent (VisualSonics, Toronto, ON, Canada). A bolus injection of 100 μl contrast agent (concentration of 2 × 10^7^ microbubbles ml^−1^) was delivered through the tail vein catheter. Acquisition parameters were kept constant at 4% power, 9 dB contrast gain, gate size of 6, high line density and wide beam width. Tumor sectional area was determined by manually outlining the borders of the tumor. Motion-compensated data analysis on stored cine loops of perfusion and imaging was performed by commercially available software (VevoCQ; VisualSonics).

### Immunofluorescence analysis of mouse tissues

To analyze vascular morphogenesis in the neonatal retina, eyes were dissected and fixed in 4% paraformaldehyde (Sigma) for 1 h on ice, washed in PBS before dissection of retinas. After blocking in TNB buffer (0.5% blocking reagent (PerkinElmer, FP1012), 150 mM NaCl, 100 mM Tris-HCl (pH 7.4), 0.4% Triton X-100) for 2 h at room temperature, the retinas were incubated with Alexa-Fluor-conjugated isolectin B4 (Invitrogen, I21411, 20 μg ml^−1^ in TNB) and primary antibody (if required) overnight at 4 °C. Anti-collagen IV (AbD Serotec, 2150–1470, 1:400), anti-ERG (Abcam, Ab92513, 1:200) or FOXO1 (Cell Signaling Technology, 2880, 1:100) and then Alexa-Fluor 546-conjugated secondary antibody (Invitrogen, 1:400) were applied in sequence. Afterwards, retinas were flat-mounted with Fluromount-G (Invitrogen) and examined by Olympus FV1000 or Zeiss LSM 710 confocal microscope. To label the proliferative endothelial cells with EdU, pups were injected i.p. with 60 μl EdU (Invitrogen, C10338, 0.5 mg ml^−1^ in PBS) 3 h before experiments. EdU detection was performed by Click-iT EdU Cell Proliferation Kit for Imaging (Invitrogen, C10338). Retinas were stained by In Situ Cell Death Detection Kit TMR red (Roche) as the manufacturer’s instruction for determination of apoptotic ECs.

In implanted tumor assays, mouse tissues were fixed in 4% paraformaldehyde overnight at 4 °C, washed in PBS, equilibrated in 30% sucrose overnight, embedded in OCT (Sakura), and sectioned at 5 μm. Slides were treated with 0.2% Triton X-100 in PBS and incubated in blocking solution (1% BSA, 5% goat serum in PBS) for 1 h at room temperature. Primary antibodies anti-CD31 (BD Biosciences, 553370, 1:200) and anti-collagen IV (AbD Serotec, 2150–1470, 1:200) were incubated overnight at 4 °C, followed with Alexa-Fluor 488- and Alexa-Fluor 546-conjugated secondary antibody (Invitrogen, 1:400) stainings for 2 h at room temperature. Samples were stained by DAPI solution for nuclei and then mounted by Fluromount-G. Slides were examined by Olympus BX-53 upright or Zeiss LSM 710 confocal microscope. Image analysis was accomplished with ImageJ. To assess tumor vessel density, CD31- positive area was measured and normalized with the tumor area. For analysis of vessel regression, the collagen IV-positive area was measured and divided by the CD31-positive area as previously reported^24^.

### Quantitative analysis of the retinal vessels

All quantifications were performed with Volocity software on high-resolution confocal images representing a thin *z* section of the retina. The sprouts, EC area, branch points and collagen IV^+^/isolectin B4^-^ sleeves were measured according to the published protocols^24,35,94^. In brief, the sprouts were defined as protrusive endothelial cells above the angiogenic front line and were quantified in a minimum of 8 fields (sized 1270 μm × 1270 μm). For each vascularized field, the proportion of EC coverage was calculated by measuring the isolectin B4-positive area normalized to the total area in a minimum of 8 fields per group. Regions of quantification were selected in 1270 μm × 1270 μm fields for EC coverage or in the vascular plexus (sized 635 μm × 635 μm) between an artery and vein excluding the angiogenic front for branching points. For the analysis of vessel stability, regions at the angiogenic front (around 400 μm × 200 μm) were selected and a minimum of 8 fields per group were taken for measurements. Vascular regression analysis was accomplished by counting collagen IV-positive and isolectin B4-negative structures and correlating them to the IB-4-positive area. For analysis of FOXO1 activation, IB4^+^/FOXO1^+^ cell nuclei were counted and correlated to the IB-4-positive area. For analysis of proliferation and apoptosis, EdU^+^/ERG^+^ nuclei or TUNEL^+^/IB4^+^ signals were counted and normalized to the vessel area. All the images shown are representative vascular phenotype observed in samples from at least two distinct litters per group.

### Retinal endothelial cell sorting

P7 retinas were dissected, minced into fragments and then digested with collagenase/dispase (Roche, 1 mg ml^−1^) in DMEM at RT for 2 min and filtered through 40 μm nylon mesh. Cell suspensions were incubated with eFluor 660-conjugated VE-cadherin antibody (eBioscience, 50-1441-82, 1:200) in 1% BSA/PBS for 1 h on ice. eFluor 660-positive endothelial cells were sorted through the instrument (BD SORP FACS Aria) and authenticated by CD31 expression.

### Quantitative real-time PCR analysis

Total RNA in sorted endothelial cells, embryos, normal or tumor tissues (grinded in liquid nitrogen) was extracted by Trizol and reverse transcribed into cDNA by RT Master Mix (TOYOBO) according to the manufacturer’s instructions. Subsequent quantitative PCR was performed with SYBR Green Real-time PCR Master Mix (TOYOBO). Gene expression was normalized to the endogenous control zebrafish *actb* or mouse *Actb*. All qPCR reactions were run on ABI VIIA7 real-time PCR instrument and data were calculated using the ΔΔ*C*_*t*_ method. Primers used for qPCR analysis were listed in Table S1.

### Western blotting analysis

Zebrafish embryos were collected at the indicated stage and directly lysed in SDS sample buffer. For nuclear FOXO1 detection, cytoplasmic and nuclear protein samples were obtained from 48 hpf zebrafish embryos using Nuclear and Cytoplasmic Protein Extraction Kit (Thermo fisher) according to the manufacturer’s instruction. Tumor tissue lysates were prepared in RIPA buffer for 30 min on ice. HUVEC samples were directly harvested into SDS sample buffer. Proteins were separated by SDS-PAGE and blotted onto polyvinylidene fluoride membranes. The following antibodies were used: anti-MYC (Genomics Technology, SG4110-18, 1:1000), anti-a-Tubulin (Sigma, T9026, 1:1000), anti-GAPDH (Absci, 21612-2, 1:2000), anti-HIF1a (Ruiying Biological, RLT2133, 1:500), anti-phospho-FOXO1 (Cell Signaling Technology, 9464, 1:500), anti-FOXO1 (Cell Signaling Technology, 2880, 1:500) and anti-Histone-3 (Cell Signaling Technology, 9715, 1:2000).

### Statistical analysis

No statistical methods were used to predetermine sample size. The experiments were not randomized and investigators were not blinded to allocation during experiments and outcome assessment. Statistical analysis was performed by Graphpad Prism 7 software. Data were analyzed by unpaired two-tailed Student’s *t*-test for two group comparison. When variances were significantly different in two groups, Welch’s correction was applied. For multiple group comparison, one-way ANOVA or two-way ANOVA (analysis of variance) followed by Bonferroni’s multiple comparison test was performed. Results were represented as mean ± SEM. *P* values <0.05 were considered as significant, *P *<* 0.05, **P < 0.01, ***P < 0.001, ****P < 0.0001 or ns (*P* ≥ 0.05). Each experiment was independently performed at least three times.

### Data availability

The data that support the findings of this study are available from the corresponding author upon reasonable request.

## Supplementary information

Supplementary information, Figure S1

Supplementary information, Figure S2

Supplementary information, Figure S3

Supplementary information, Figure S4

Supplementary information, Figure S5

Supplementary information, Figure S6

Supplementary information, Figure S7

Supplementary information, Figure S8

Supplementary information, Table S1

Supplementary information, Supplementary video legends

Supplementary information, Supplementary Video S1

Supplementary information, Supplementary Video S2

Supplementary information, Supplementary Video S3

Supplementary information, Supplementary Video S4
