# Supplementary material for: Endothelial CDS2 deficiency causes VEGFA-mediated vascular regression and tumor inhibition
Source: Cell Res. 2019 Sep 9;29(11):895–910. doi: 10.1038/s41422-019-0229-5 (PMC6889172; doi:10.1038/s41422-019-0229-5)
Supplement: Supplementary file 1 — Supplementary information, Figure S1 [file 41422_2019_229_MOESM1_ESM.pdf]

Supplementary information, Figure S1

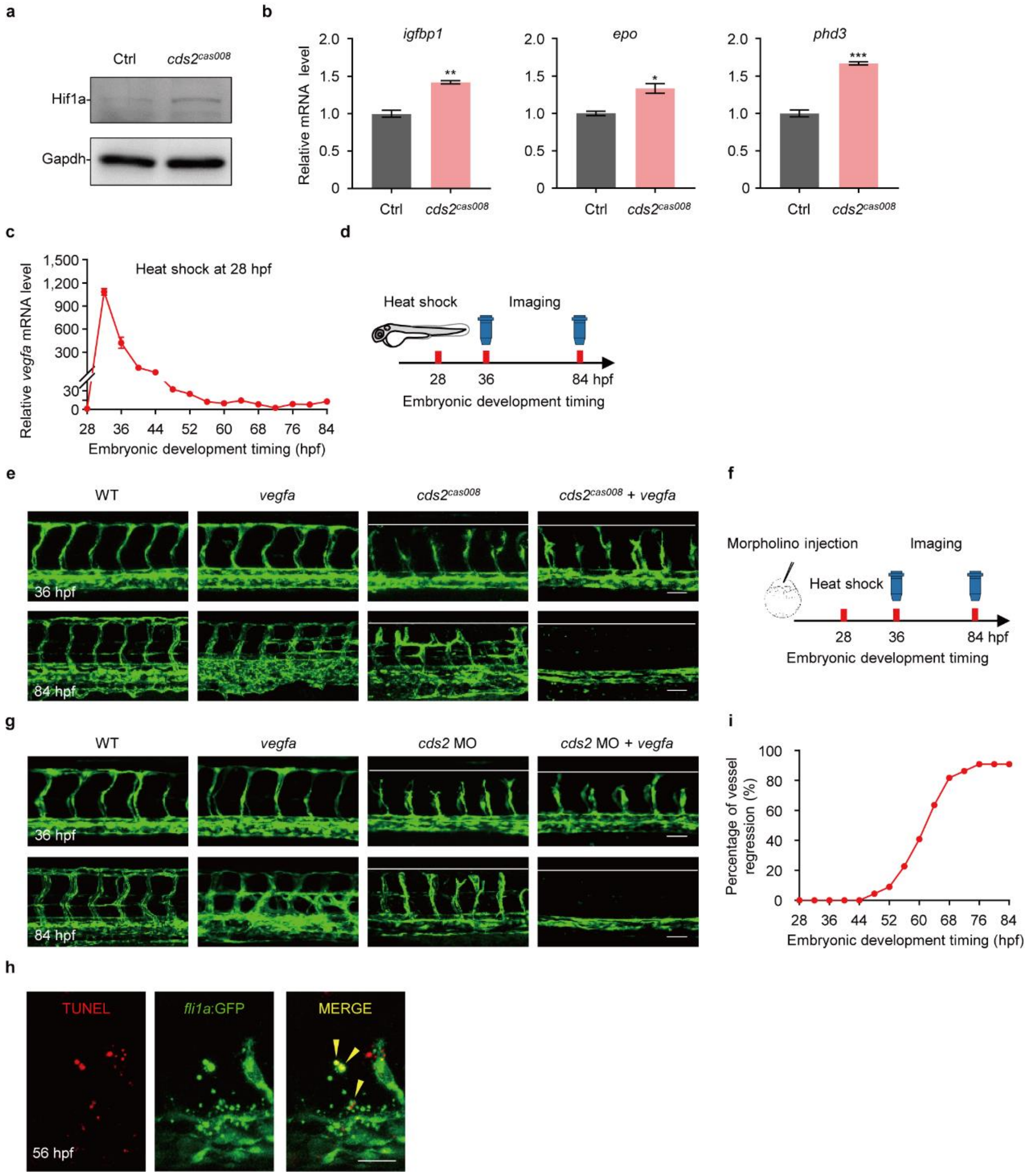

Fig. S1. VEGFA induces vessel regression in zebrafish *cds2* mutants. (a, **b)** Western blotting analysis of Hif1a protein level (**a**) and relative expression of Hif target genes (**b**) in WT and *cds2* mutant embryos (severe phenotype) at 36 hpf. n = 3 samples, 3-5 embryos pooled for each sample (**b**). **(c)** Relative *vegfa* expression at different time points after heatshock at 28 hpf. *vegfa* level was normalized to wild type embryos in the same experimental conditions. n = 3 samples per point, 10 embryos pooled for each sample. **(d)** Model of timing for heatshock induction and confocal imaging analysis in (Fig. S1**e**). **(e)** Confocal images of trunk vessels in WT and *cds2* mutant under *Tg(fli1a:eGFP)* background, with or without *vegfa* overexpression (OE) at 36 hpf and 84 hpf. *vegfa* OE was induced by heat shock at 28 hpf in *Tg(hsp:vegfaa)* background. **(f)** Model of timing for morpholino injection (MI), heatshock induction and confocal imaging analysis in (Fig. S1**g**). **(g)** Confocal images of trunk vessels in WT and *cds2* MO-injected embryos with or w/o *vegfa* OE under *Tg(fli1a:eGFP)* background. **(h)** Representative confocal images of TUNEL staining in 56 hpf *cds2* mutants with *vegfa* OE during vessel regression. **(i)** Time-lapse phenotype analysis of vessel regression in *cds2* morphants with *vegfa* OE. n = 300 ISVs counted from 30 embryos per group.Scale bars, 50 μm (**c** and **e**; 36 hpf), 70 μm (**c** and **e**; 84 hpf) and 25 μm (**h**). Error bars, mean ± SEM. *$P < 0.05$; **$P < 0.01$; ***$P < 0.001$.
