# Supplementary material for: Endothelial CDS2 deficiency causes VEGFA-mediated vascular regression and tumor inhibition
Source: Cell Res. 2019 Sep 9;29(11):895–910. doi: 10.1038/s41422-019-0229-5 (PMC6889172; doi:10.1038/s41422-019-0229-5)
Supplement: Supplementary file 2 — Supplementary information, Figure S2 [file 41422_2019_229_MOESM2_ESM.pdf]

Supplementary information, Figure S2

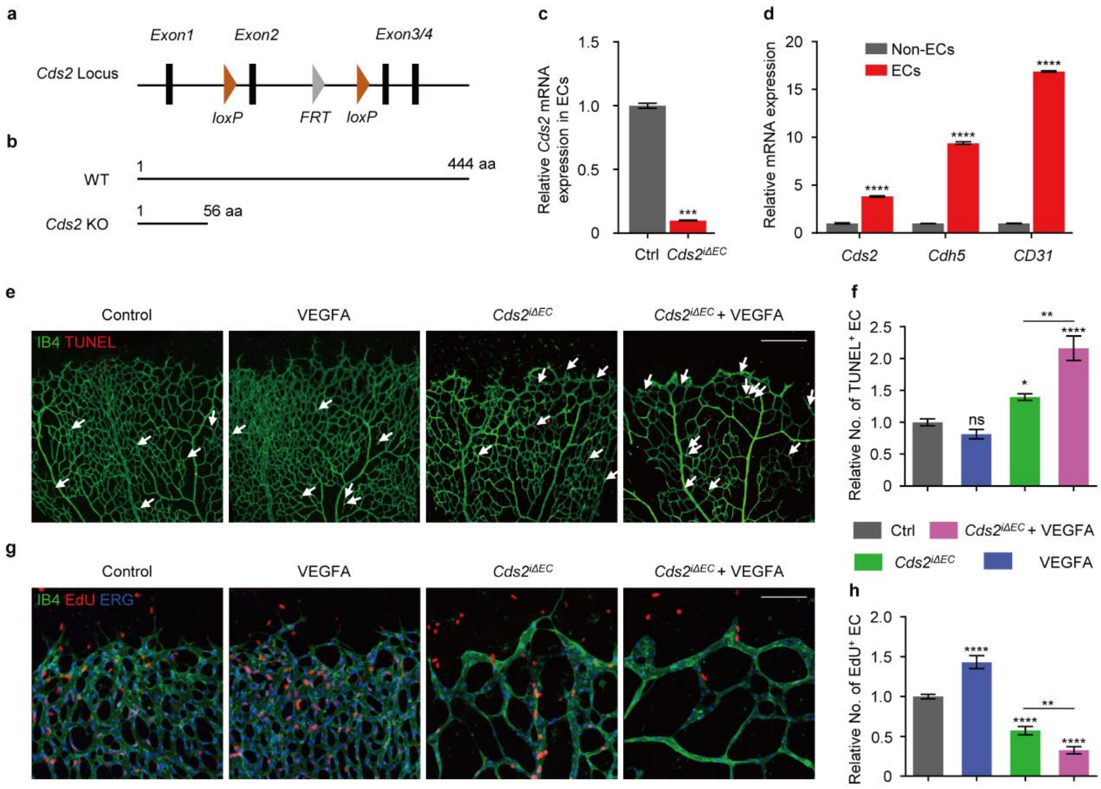

Fig. S2. Decreased cell proliferation and increased cell apoptosis involved in vessel regression. (a, **b)** Strategic diagram of *Cds2* conditional knockout allele in which exon 2 is flanked by loxp sites (**a**). After Cre activation, recombination results in a deletion of exon 2 and creates a stop codon to generate a premature protein at amino acid 56 (**b**). **(c**, **d)** Quantitative RT-PCR analysis of *Cds2* mRNA level (**c**) normalized to *Actb* in isolated endothelial cells. *Cdh5* and *CD31* (**d**) were used as positive controls to confirm the enrichment of *Cds2* in endothelium. *n* = 3 experiments. **(e)** Retinal vasculature stained with IB4 and by TUNEL assay in P7 $Cds2^{i\Delta EC}$ or control mice with or w/o VEGFA injection. Apoptotic ECs are indicated by arrows. **(f)** Analysis of relative number of apoptotic ECs in (**e**). *n* = 8 mice per group. **(g)** Staining of retinal vasculature by IB4, EdU and ERG in the angiogenic front of P7 $Cds2^{i\Delta EC}$ or control mice with or w/o VEGFA injection. **(h)** Analysis of relative number of cell proliferation in (**g**). *n* = 6 mice per group. Scale bars, 300 μm (**e**) and 100 μm (**g**). Error bars, mean ± SEM. *P < 0.05; **P < 0.01; ***P < 0.001; ****P< 0.0001; ns, not significant ($P \geq 0.05$).
