# Supplementary material for: Endothelial CDS2 deficiency causes VEGFA-mediated vascular regression and tumor inhibition
Source: Cell Res. 2019 Sep 9;29(11):895–910. doi: 10.1038/s41422-019-0229-5 (PMC6889172; doi:10.1038/s41422-019-0229-5)
Supplement: Supplementary file 3 — Supplementary information, Figure S3 [file 41422_2019_229_MOESM3_ESM.pdf]

Supplementary information, Figure S3

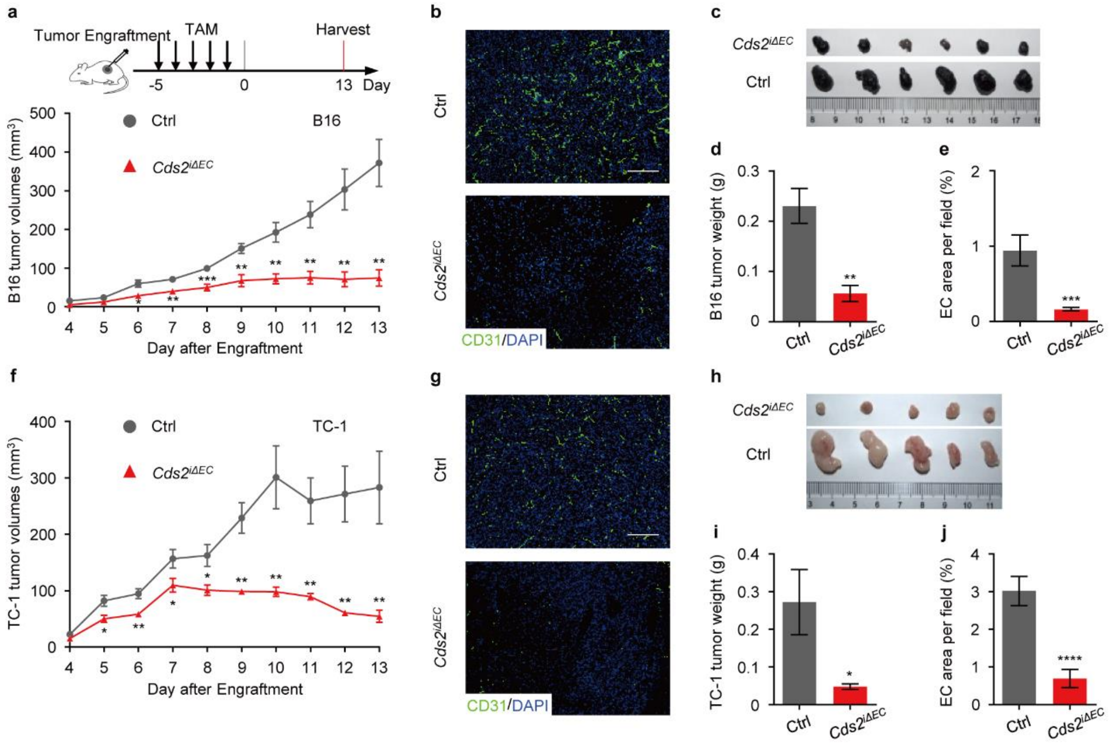

Fig. S3. *Cds2* EC-specific KO blocks tumor growth and angiogenesis. (a-e) EC-specific ablation of *Cds2* (**a**) resulted in limited B16 tumor growth (**a**), decreased vessel area (**b** and **e**), reduced tumor size (**c**) and weight (**d**). Quantification of vessel density is shown in (**e**). Schematic diagram (**a**) shows the strategy for genetic ablation of *Cds2* before tumor implantation. TAM, tamoxifen. n = 6-8 tumors from 6-8 mice per group. **(f-j)** Genetic depletion of *Cds2* in ECs (**a**) caused limited TC-1 tumor growth (**f**), decreased vessel area (**g** and **j**) and reduced tumor size (**h**) and weight (**i**). Vessel density was quantified in (**j**). n = 5-8 tumors from 5-8 mice per group. Scale bars, 200 μm. Error bars, mean ± SEM. *$P < 0.05$; **$P < 0.01$; ***$P < 0.001$; ****$P < 0.0001$; ns, not significant ($P \geq 0.05$).
