# Supplementary material for: Endothelial CDS2 deficiency causes VEGFA-mediated vascular regression and tumor inhibition
Source: Cell Res. 2019 Sep 9;29(11):895–910. doi: 10.1038/s41422-019-0229-5 (PMC6889172; doi:10.1038/s41422-019-0229-5)
Supplement: Supplementary file 4 — Supplementary information, Figure S4 [file 41422_2019_229_MOESM4_ESM.pdf]

Supplementary information, Figure S4

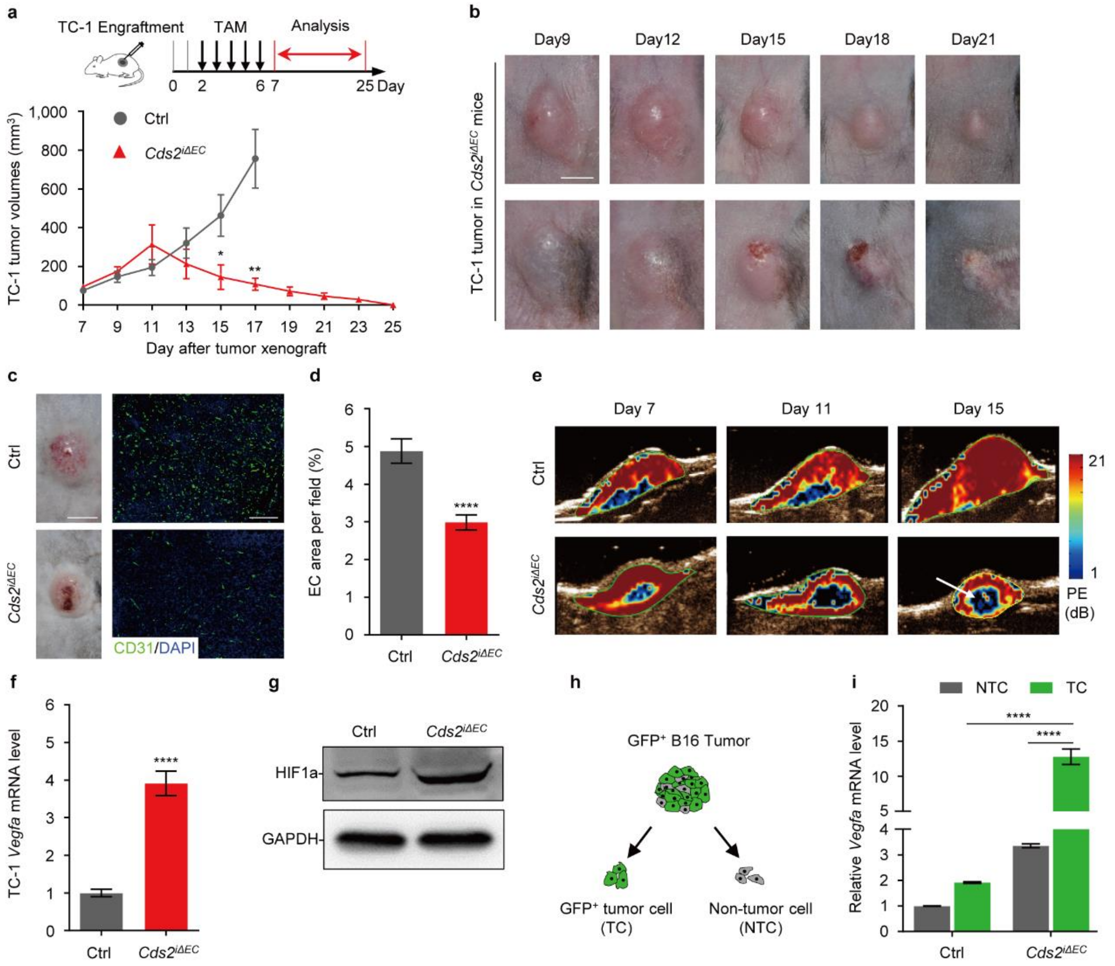

Fig. S4. Endothelial deprivation of *Cds2* causes tumor vessel regression. (a-e) Inactivation of *Cds2* in ECs (**a**) blocks TC-1 tumor growth (**a**), causes tumor regression (**b**) or necrosis (**c**; left panels, day 11 tumors), reduces vessel density (**c**; right panels and **d**), and results in poor blood perfusion (**e**; arrow shows severely reduced blood flow in TC-1 tumors). Schematic diagram (**a**) shows the strategy to inactivate *Cds2* after tumor implantation. Tumor regression and necrosis were monitored from day 7-25. A portion of tumors were collected for immunofluorescence at day 13, or administrated for ultrasound analysis at day 7, 11 and 15. n = 6 tumors from 6 mice per group. **(f)** Quantification of *Vegfa* level in day 9 TC-1 tumors. n = 8 tumors from 4 mice per group. **(g)** Western blotting analysis of HIF1a protein level in day 9 B16 tumors from control and $Cds2^{i\Delta EC}$ mice. (**h**, **i**) Relative *Vegfa* expression level in day 9 B16 tumor cells and non-tumor cells from control or $Cds2^{i\Delta EC}$ mice. Cartoon model shows the cell sorting strategy of GFP-labeled B16 tumor and non-tumor cells (**h**). n = 3 samples from 8 tumors of 4 mice each group. Scale bars, 0.5 cm (**b** and **c**; left panel) and 200 μm (**c**; right panel). Error bars, mean ± SEM. *P < 0.05; **P < 0.01; ****P < 0.0001; ns, not significant ($P \geq 0.05$).
