# Supplementary material for: Endothelial CDS2 deficiency causes VEGFA-mediated vascular regression and tumor inhibition
Source: Cell Res. 2019 Sep 9;29(11):895–910. doi: 10.1038/s41422-019-0229-5 (PMC6889172; doi:10.1038/s41422-019-0229-5)
Supplement: Supplementary file 5 — Supplementary information, Figure S5 [file 41422_2019_229_MOESM5_ESM.pdf]

Supplementary information, Figure S5

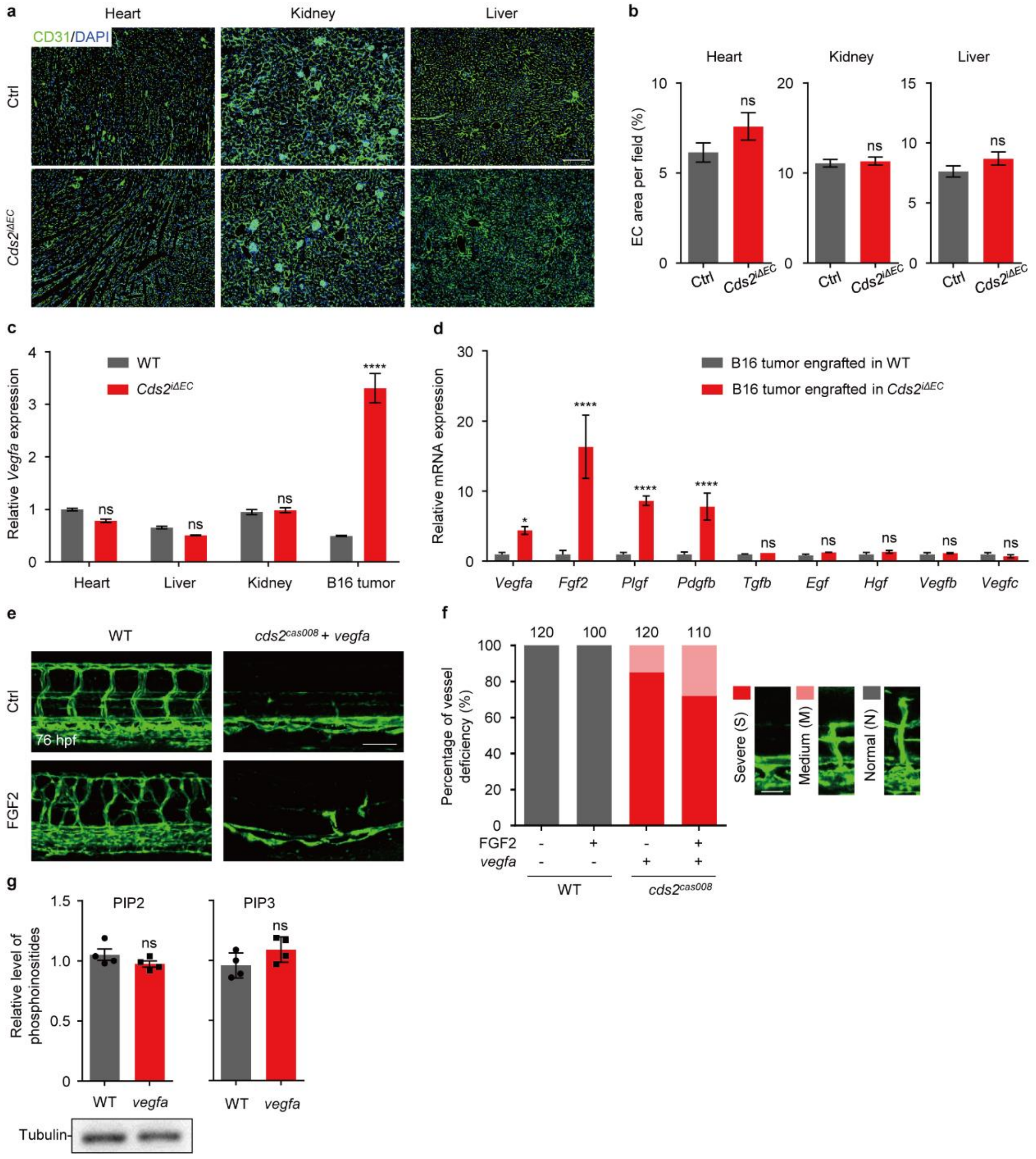

Fig. S5. Vasculature in organs of heart, liver and kidney from *Cds2*$^{i\Delta EC}$ mice is normal. (a, b) Images (**a**) and quantitative analysis (**b**) of CD31 staining of heart, kidney and liver from control and *Cds2*$^{i\Delta EC}$ mice. n = 7-9 mice per group. **(c)** Relative *Vegfa* expression levels of B16 tumors and organs such as heart, liver and kidney in control and *Cds2*$^{i\Delta EC}$ mice. n = 3 samples from 6 mice per group. **(d)** Relative expression levels of pro-angiogenic factors in day13 B16 tumors from control and *Cds2*$^{i\Delta EC}$ mice. n = 6 tumors from 6 mice per group. **(e, f)** Confocal images (**e**) and quantitative analysis (**f**) of zebrafish trunk vessels show ectopic FGF2 could not rescue vessel regression in *cds2* mutant embryos with *vegfa* OE. FGF2 protein was delivered through venous injection at 28-30 hpf, which caused hyper-branching of ISV in control embryos. Zebrafish embryos were imaged at 76 hpf. The representative images of classified recovery phenotypes are shown on the right (**f**). The quantified ISV number shown on the top graph (**f**) is from 10-12 embryos per group. **(g)** Quantification of phosphoinositides in the endothelium from 48 hpf control or *vegfa* OE embryos. Western blotting analysis on a-Tubulin serves as the internal control for cell amounts. n = 4 samples per group. Scale bars, 200 μm (**a**), 100 μm (**e**) and 50 μm (**f**). Error bars, mean ± SEM. *$P < 0.05$; ****$P < 0.0001$; ns, not significant ($P \geq 0.05$).
