# Supplementary material for: Endothelial CDS2 deficiency causes VEGFA-mediated vascular regression and tumor inhibition
Source: Cell Res. 2019 Sep 9;29(11):895–910. doi: 10.1038/s41422-019-0229-5 (PMC6889172; doi:10.1038/s41422-019-0229-5)
Supplement: Supplementary file 6 — Supplementary information, Figure S6 [file 41422_2019_229_MOESM6_ESM.pdf]

Supplementary information, Figure S6

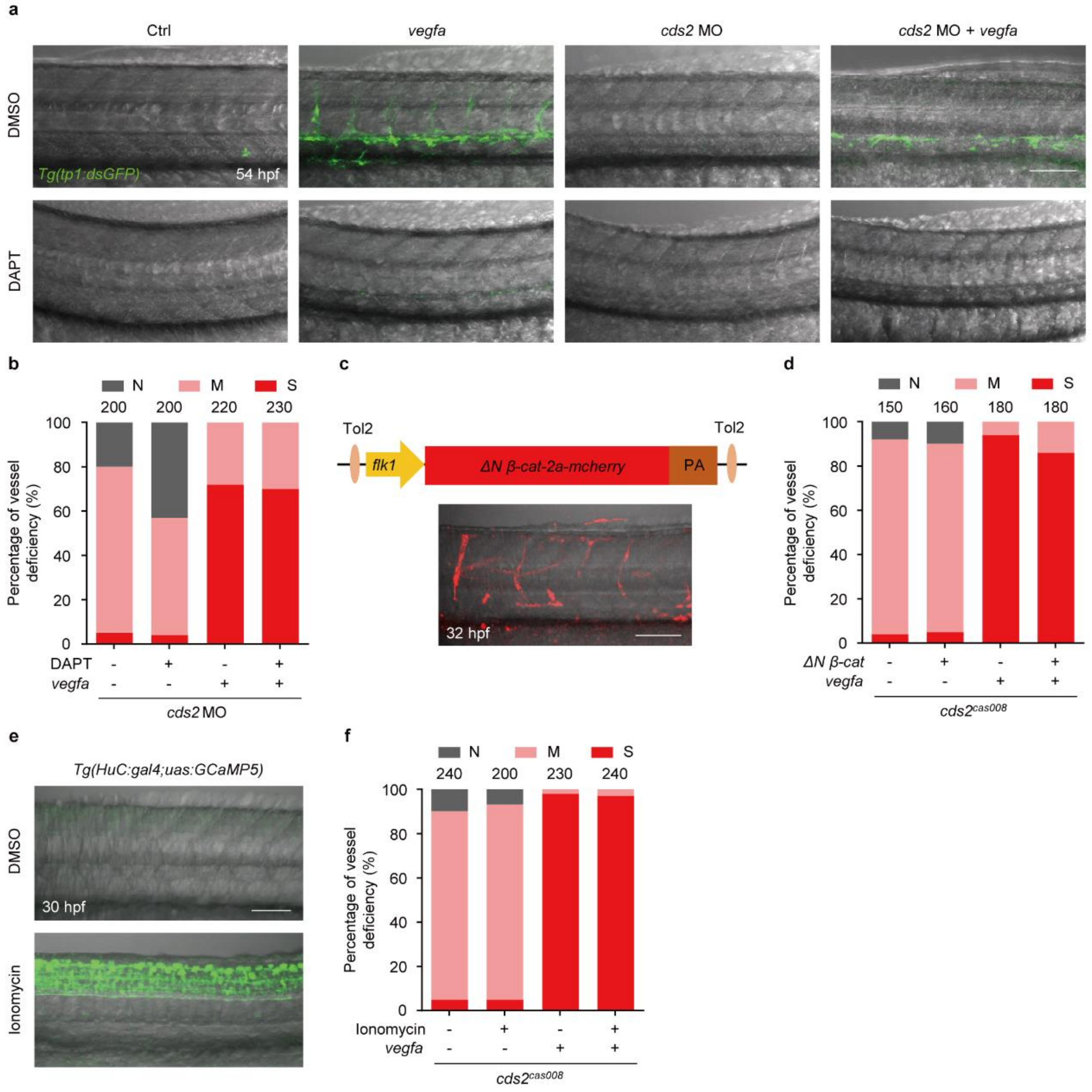

Fig. S6. Cell signaling analysis of VEGFA-induced vessel regression in CDS2-deficient endothelium. (a) Confocal images of trunk regions from 54 hpf embryos of control, *vegfa* OE, *cds2* morphants and *cds2* morphants with *vegfa* OE harboring *Tg(tp1:dsGFP)* background treated with DAPT (Notch inhibitor) or DMSO. DAPT or DMSO was administrated from 24 hpf to the indicated stage. **(b)** Quantification of vessel deficiency shows inhibition of Notch signaling by DAPT couldn't rescue vessel regression. N, normal, M, medium, S, severe. The quantified ISV number shown on the top is from 20-23 embryos per group. **(c)** Construction of *Tol2:flk1-ΔN β-cat-2a-mcherry* for transgenesis. The construct was injected together with transposase mRNA into WT or *cds2* mutant embryos at 1-2 cell stage. Confocal image taken at 32 hpf confirmed the efficiency and specificity of transient transgenesis. **(d)** Quantitative analysis of the rescue effects of activated Wnt/β-catenin signaling on VEGFA-induced vessel regression. *ΔN β-cat*, *Tol2:flk1-ΔN β-cat-2a-mcherry* mediated transgenesis. The counted ISV number is from 15-18 embryos per group. **(e)** Ca^{2+} signaling activation by ionomycin treatment. 30 hpf zebrafish embryos within *Tg(HuC:gal4,uas:GCaMP5)* background were treated with 500 nM ionomycin at 30 hpf for 10 minutes, followed by imaging analysis. **(f)** Quantitative analysis of rescue effects of ionomycin treatment on VEGFA-induced vessel regression. Ionomycin treatment was performed from 24 to 76 hpf. The counted ISV number is from 20-24 embryos per group. Scale bars, 100 μm.
