# Supplementary material for: Endothelial CDS2 deficiency causes VEGFA-mediated vascular regression and tumor inhibition
Source: Cell Res. 2019 Sep 9;29(11):895–910. doi: 10.1038/s41422-019-0229-5 (PMC6889172; doi:10.1038/s41422-019-0229-5)
Supplement: Supplementary file 7 — Supplementary information, Figure S7 [file 41422_2019_229_MOESM7_ESM.pdf]

Supplementary information, Figure S7

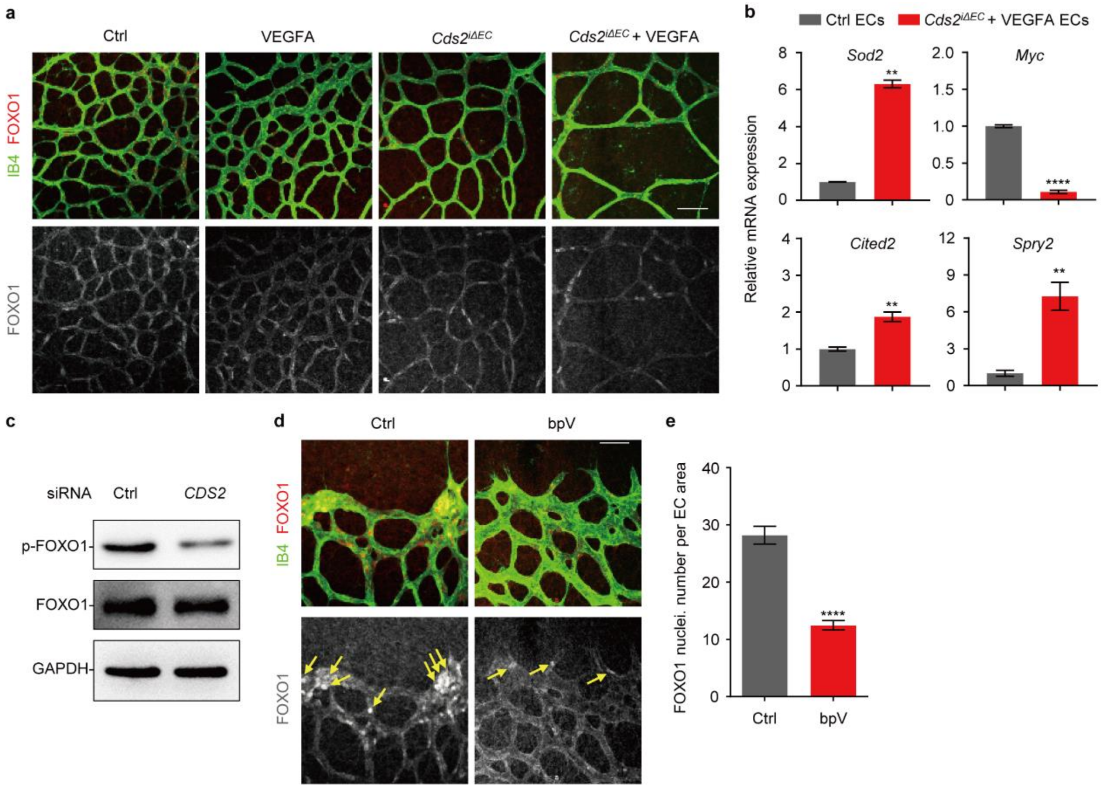

Fig. S7. FOXO1 activation is required for VEGFA-induced vessel regression of CDS2-deficient endothelium. (a) Confocal images of IB4 and FOXO1 co-staining of P7 retinal vessels in the remodeling plexus area from WT or *Cds2*$^{i\Delta EC}$ embryos with or without ectopic VEGFA injection. **(b)** Transcript analysis of FOXO1 target genes in retinal ECs from P7 control or *Cds2*$^{i\Delta EC}$ mice with VEGFA injection. FOXO1 upregulated genes: *Sod2*, *Cited2*, *Spry2*; downregulated gene: *Myc*. n = 3 samples from 6 mice per group. **(c)** phospho-FOXO1 determination by western blotting in HUVECs transfected with control or *CDS2* siRNA. **(d**, **e)** Confocal images (**d**) and quantitative analysis (**e**) of IB4 and FOXO1 co-immunostaining of P7 retinas from VEGFA-injected *Cds2*$^{i\Delta EC}$ mice treated with vehicle or small molecular compound bpV. Yellow arrows indicate nuclear accumulation of FOXO1. Ctrl, VEGFA-injected *Cds2*$^{i\Delta EC}$ mice treated with saline (vehicle for bpV). n = 8 mice per group. Scale bars, 50 μm. Error bars, mean ± SEM. $^{**}P < 0.01$; $^{****}P < 0.0001$.
