# Supplementary material for: Endothelial CDS2 deficiency causes VEGFA-mediated vascular regression and tumor inhibition
Source: Cell Res. 2019 Sep 9;29(11):895–910. doi: 10.1038/s41422-019-0229-5 (PMC6889172; doi:10.1038/s41422-019-0229-5)
Supplement: Supplementary file 8 — Supplementary information, Figure S8 [file 41422_2019_229_MOESM8_ESM.pdf]

Supplementary information, Figure S8

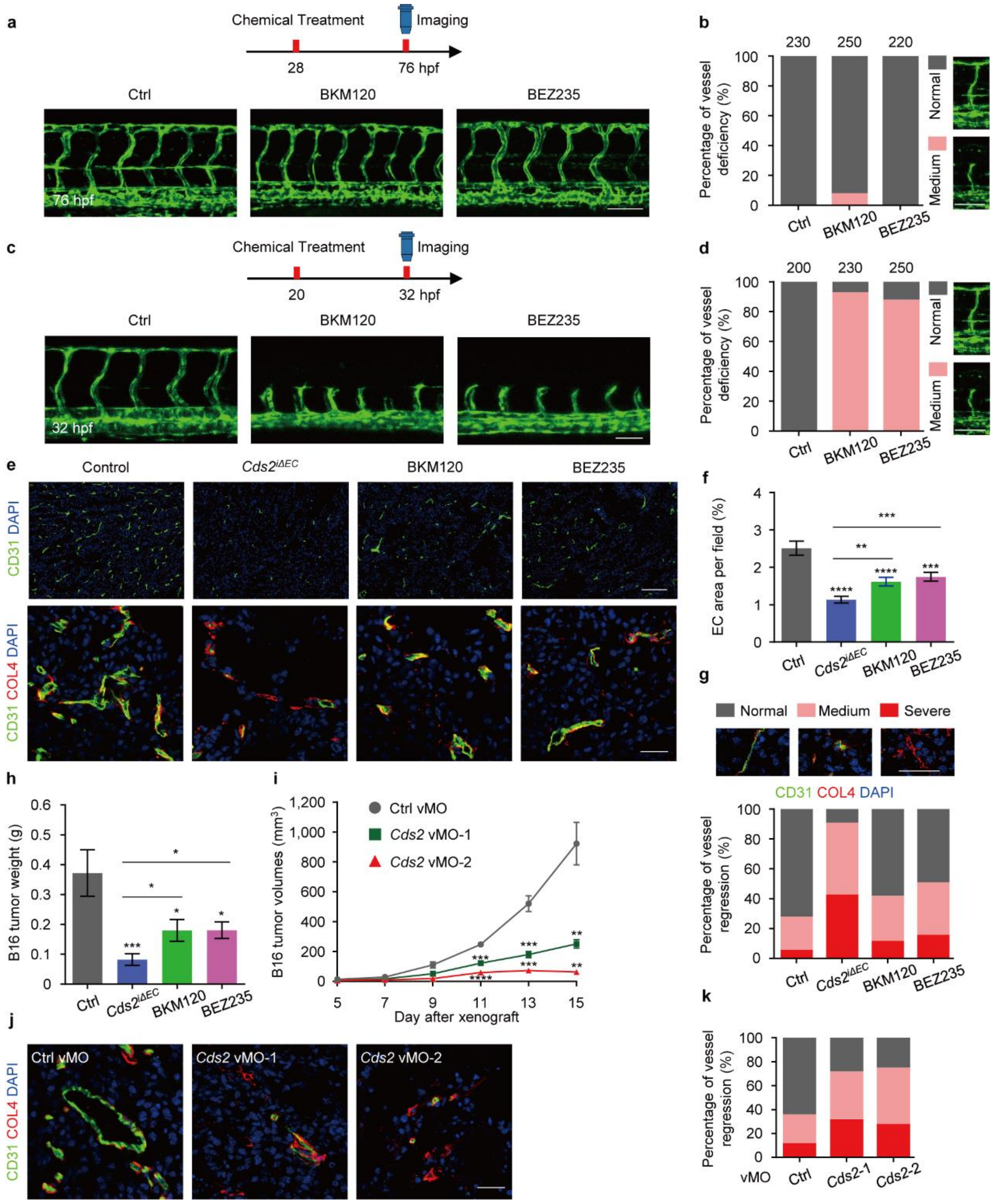

Fig. S8. PIP3 perturbation is not sufficient to trigger vessel regression. (a, b) Confocal images (**a**) and quantification (**b**) of trunk vessel phenotypes from zebrafish embryos treated with ctrl (DMSO) or PI3K inhibitors (BKM120 and BEZ235) from 28-76 hpf. The diagram (**a**) shows the timing for chemical treatment. The quantified ISV phenotypes were shown on the right (**b**). The ISV numbers counted on the top (**b**) were from 22-25 embryos. **(c, d)** Confocal images (**c**) and quantification (**d**) of trunk vessel phenotypes in zebrafish embryos with ctrl (DMSO) or PI3K inhibitor (BKM120 and BEZ235) treatment. Chemicals were added from 20 hpf until the indicated stage (**c**). The ISV numbers counted on the top (**d**) were from 20-25 embryos. **(e, f)** Images (**e**; top panel) and quantification (**f**) of $CD31^+$ endothelium in B16 tumors from $Cds2^{i\Delta EC}$ mice or PI3K inhibitor-treated WT mice at day 10 post-implantation. *n* = 12 tumors from 12 mice per group. **(e, g)** Confocal images (**e**; bottom panel) and quantification (**g**) of vessel regression ($COL4^+$ $CD31^-$) in B16 tumors from $Cds2^{i\Delta EC}$ mice or PI3K inhibitor-treated WT mice at day 10 post-implantation. Regressed vessel is assessed as normal, medium and severe, the representative images are shown on the top panel (**g**). Counted vessel number $n \geq 150$ per group, 36 fields were counted from 12 tumors in 12 mice each group. **(h)** Quantification of B16 tumor weight in $Cds2^{i\Delta EC}$ mice or PI3K inhibitor-treated WT mice. *n* = 6 tumors from 6 mice per group. **(i-k)** Administration of *Cds2* vivo-morpholino in WT mice results in limited B16 tumor growth (**i**) and induces tumor vascular regression (**j**, **k**). *n* = 6 mice each group. Scale bars, 100 μm (**a**, **b** and **d**), 50 μm (**c**, **e** (bottom panel), **g**, and **j**) and 200 μm (**e**; top panel). Error bars, mean ± SEM. *$P < 0.05$; **$P < 0.01$; ***$P < 0.001$; ****$P < 0.0001$.
