# Supplementary material for: Endothelial CDS2 deficiency causes VEGFA-mediated vascular regression and tumor inhibition
Source: Cell Res. 2019 Sep 9;29(11):895–910. doi: 10.1038/s41422-019-0229-5 (PMC6889172; doi:10.1038/s41422-019-0229-5)
Supplement: Supplementary file 10 — Supplementary information, Supplementary video legends [file 41422_2019_229_MOESM10_ESM.docx]

**Supplementary Video Legends**

**Video S1.** **Vessel regression in *cds2* mutants with *vegfa* overexpression**.

Trunk vasculature (*fli1a:eGFP*) of *cds2* mutant embryo with *vegfa* OE was imaged from 52-72 hpf. Endothelial cells migrated reversely to the axial vessels and partially underwent burst before merging into axial vessels. Time-lapse live imaging was captured at 12 minutes/frame and rendered at 10-frames/s. Scale bar, 50 μm.

**Video S2. Vessel regression in *cds2* mutants with endogenous *vegfa*.**

Trunk vasculature (*fli1a:eGFP*) of *cds2* mutant embryo was imaged from 36-52 hpf. Vessel regression stopped around 52 hpf with ISVs remains to myoseptum. Time-lapse live imaging was captured at 12 minutes/frame and rendered at 5-frames/s. Scale bar, 50 μm.

**Video S3.** **Endothelial cell proliferation in normal angiogenesis.**

Trunk vasculature (*fli1a:ngfp*) of WT embryo was imaged from 23-42 hpf. EC proliferation was frequently occurred during angiogenesis. Time-lapse live imaging was captured at 14 minutes/frame and rendered at 10-frames/s. Scale bar, 40 μm.

**Video S4. Enhanced apoptosis but impaired proliferation in *cds2*-deficient endothelium with *vegfa* OE.**

Trunk vasculature (*fli1a:ngfp*) of *cds2* MO-injected embryo with *vegfa* OE was imaged from 52-72 hpf. EC reverse migration and apoptosis were implicated in vessel regression, while cell proliferation barely occurred in this process. Time-lapse live imaging was captured at 14 minutes/frame and rendered at 10-frames/s. Scale bar, 40 μm.
